# Large-scale longitudinal gradients of genetic diversity: a meta-analysis across six phyla in the Mediterranean basin

**DOI:** 10.1002/ece3.350

**Published:** 2012-09-14

**Authors:** Cyrille Conord, Jessica Gurevitch, Bruno Fady

**Affiliations:** 1 INRA, FR ECCOREV, UR629, Écologie des Forêts Méditerranéennes84914, Avignon, France; 2 Laboratoire BVpam, Université de Saint-Etienne, Jean MonnetEA2061, 23 rue du Dr Michelon, 42000, Saint-Etienne, France; 3 Department of Ecology and Evolution, Stony Brook UniversityStony Brook, New York, 11794–5245

**Keywords:** Arthropod, biodiversity, biogeography, Bryophyte, Chordata, genetic diversity, Holocene, longitude, meta-analysis, Mollusc, past climate, phylogeography, Pleistocene, Pteridophyte, recolonization, Spermaphyte

## Abstract

Biodiversity is the diversity of life at all scales, from genes to ecosystems. Predicting its patterns of variation across the globe is a fundamental issue in ecology and evolution. Diversity within species, that is, genetic diversity, is of prime importance for understanding past and present evolutionary patterns, and highlighting areas where conservation might be a priority. Using published data on the genetic diversity of species whose populations occur in the Mediterranean basin, we calculated a coefficient of correlation between within-population genetic diversity indices and longitude. Using a meta-analysis framework, we estimated the role of biological, ecological, biogeographic, and marker type factors on the strength and magnitude of this correlation in six phylla. Overall, genetic diversity increases from west to east in the Mediterranean basin. This correlation is significant for both animals and plants, but is not uniformly expressed for all groups. It is stronger in the southern than in the northern Mediterranean, in true Mediterranean plants than in plants found at higher elevations, in trees than in other plants, and in bi-parentally and paternally than in maternally inherited DNA makers. Overall, this correlation between genetic diversity and longitude, and its patterns across biological and ecological traits, suggests the role of two non-mutually exclusive major processes that shaped the genetic diversity in the Mediterranean during and after the cold periods of the Pleistocene: east-west recolonization during the Holocene and population size contraction under local Last Glacial Maximum climate in resident western and low elevation Mediterranean populations.

## Introduction

Biodiversity is the diversity of life at all scales, that is, “the variability among living organisms from all sources including, *inter alia*, terrestrial, marine and other aquatic ecosystems and the ecological complexes of which they are part; this includes diversity within species, between species and of ecosystems” (article 2 of the Convention of Biological Diversity 1992). Although knowledge of the distribution of species is far from always being spatially accurate and detailed (Richardson and Whittaker 2010), species diversity and abundance is relatively well known for several taxonomic groups (e.g., mammals, birds, fishes, vascular plants) and particularly in the temperate regions of the world. This knowledge has helped shaping the delineation of hotspots of biological diversity (Myers et al. 2000) where conservation is most critical and is at the core of the field of conservation biogeography (Whittaker et al. 2005).

At a finer taxonomic scale, genetic diversity, diversity among individuals within species, yields valuable information for understanding how biodiversity changes within an evolutionary framework. Within the broad context of biogeography, genetic diversity has been assessed either through ecogeographic or through phylogeographic perspectives (Avise 2000). The ecogeographic view focuses on patterns produced by contemporary natural selection, as for example, the genetic structure of Mediterranean pine stands exposed to wild fires (Aravanopoulos et al. 2004). Conversely, the phylogeographic approach focuses largely on historical evolutionary processes, such as the balance between vicariance and dispersal to examine genetic differentiation both among and within populations. Measures of genetic differentiation among populations, mapped against major geographic barriers, have been used to derive the most likely Quaternary glacial refugia and Holocene recolonization routes of major temperate species (Taberlet et al. 1998; Hewitt 1999). Measures of genetic diversity within population (*GDpop*) have been used to refine how Holocene recolonization occurred for multiple species and vegetation types, for example, from southern refugia in Europe and North America (Petit et al. 2003; Soltis et al. 2006).

Large-scale phylogeographic studies have identified latitudinal Holocene recolonization as well as complex patterns of post-glaciation dispersal from refugia at landscape to regional scales as the major drivers of genetic diversity in the Northern Hemisphere (e.g., Brewer et al. 2002; Petit et al. 2003; Liepelt et al. 2009; for Europe). Phylogeographic studies at large scales have not often considered longitude as a potentially important ecological driver, even though refugia are distributed longitudinally in southern Europe (Stewart et al. 2010). In the Mediterranean basin, longitudinal trends are potentially an important factor in determining genetic diversity because of how the geography of southern Europe and the Mediterranean region is shaped. The Mediterranean Sea is a strong barrier to latitudinal movements of terrestrial species, but also to longitudinal movements from one peninsula to the other in Europe, with potentially strong impacts in shaping contemporary biodiversity structures. Unveiling longitudinal patterns of diversity in the Mediterranean would be of great interest because, quoting from Atkinson et al. (2007), “longitudinal processes represent the raw material on which later latitudinal processes work” in Europe. The purpose of this study is to examine whether longitudinal patterns of genetic diversity are important in the Mediterranean region and southern Europe across a large range of taxa. Such patterns may reveal a different perspective on post-glaciation colonization at large geographic and taxonomic scales.

In the same manner that a gene tree only depicts a very small part of the phylogenetic history of lineage, the population genetic structure of species can only represent a small slice of the history of a whole region or biome. Whether they have followed the ecogeographic or the phylogeographic approach, many empirical studies of population genetic structure provide *GDpop* estimates. Gathering and comparing the wealth of information contained in these empirical studies, using a proper statistical framework, represents an excellent opportunity to meet the challenge of testing processes determining genetic diversity patterns at regional scale or biome-wide, such as for the Mediterranean. We used meta-analysis to document and test the existence of cross-taxa longitudinal patterns of genetic diversity in the Mediterranean basin. No previous studies have examined genetic differentiation within and across populations at large geographic scales, using the powerful statistical tools of meta-analysis.

Biogeographic genetic analyses have mostly focused on population structure and differentiation rather than on within-population diversity because genetic variation at neutral markers is not expected to respond to environmental effects (but see, e.g., Petit et al. 2003). Strong spatial gradients of neutral genetic differentiation are thus only expected as a consequence of historical effects such as directional dispersal during range expansions from refugia during global warming periods, which leads to marked population structure (Petit et al. 2003). However, because demographic changes can impact *GDpop* (Young et al. 1996), strong gradients of *GDpop* can also be expected as an indirect response to clinal environmental effects, such as past climates.

*GDpop* is of fundamental importance in ecology and evolution because it is correlated with population demographic rates and, in numerous circumstances, with their potential for evolutionary adaptive change (Le Corre and Kremer 2003). *GDpop* is therefore important for identifying regions where evolutionary potential is either particularly low or high, thus providing insights for conservation strategies and planning (Schwartz et al. 2007). *GDpop* can be estimated in various ways, but the approaches fall within two general categories: “richness” (total amount of diversity, e.g., allelic richness, haplotypic richness) and “equitability” (the way diversity is distributed among samples, e.g., heterozygosity, Shannon's index, percentage of polymorphic loci). Whereas equitability measures are more sensitive to higher frequency alleles and how they are distributed within populations, richness measures respond more to the presence and quantity of rare alleles. Thus, the two measures are needed concurrently to document and test for demographic events, such as bottlenecks and expansions.

The Mediterranean basin is a hotspot of species diversity (Myers et al. 2000), and also a world region of unusually high *GDpop* (see Fady 2005 for conifers). Vascular plants are structured into regional hotspots of species diversity and endemism (Médail and Quézel 1997) often corresponding to glacial refugia (Médail and Diadema 2009). The northern Mediterranean basin is made of south-north oriented peninsulas identified as independent Quaternary glacial refugia and starting points of Holocene recolonization for Europe (Hewitt 1999; Petit et al. 2003). The shoreline of the southern Mediterranean basin is more or less linear, without major peninsulas. Its western part, North Africa, is also recognized as a refugial zone (e.g., Cheddadi et al. 2009; Guzmán and Vargas 2009).

Two major causes can be hypothesized for longitudinal trends of *GDpop*, if such trends can be demonstrated, for natural populations in the Mediterranean (both in southern Europe and North Africa). First, longitudinal trends could result from genetic drift due to long distance dispersal and founder effects during Holocene recolonization from refugia (e.g., from eastern Mediterranean refugia as in the tree *Pinus halepensis*, Grivet et al. 2009; or in the wasp *Andricus quercustozae*, Rokas et al. 2003; from western Mediterranean refugia as in the tree *Pinus sylvestris*, Soranzo et al. 2000). However, both uni-directional recolonization patterns appear less likely than multi-refugium recolonization patterns (Taberlet et al. 1998). The second cause for longitudinal trends in this region could be genetic drift due to decreasing effective population size, given the existence of a climate of increasing severity from east to west in the Mediterranean during the last glacial cycle, particularly the Last Glacial Maximum (LGM) 21 000 years before present (van Andel 2002; Wu et al. 2007). There is also evidence of climatic instability over the North-Atlantic Ocean leading to several extreme cooling events over the Iberian Peninsula during the last glacial cycle (Sánchez-Goñi et al. 2002). The potential effect of such past climate trends on *GDpop* was described for gallwasps (Atkinson et al. 2007) and trees (Fady and Conord 2010).

Looking at patterns across multiple levels of biodiversity provides a framework to understand processes beyond the idiosyncrasy of case studies. Here, using a meta-analytical framework, we tested the existence of a longitudinal trend of within-population genetic diversity in the Mediterranean basin at multiple taxonomic levels in the tree of life. For each population genetic study we retrieved from the literature, we calculated a correlation coefficient between longitudinal coordinates and genetic diversity. We examined data on populations in the Arthropods, Mollusks, Chordata, Bryophytes, Pteridophytes, and Spermaphytes. Previous studies of genetic diversity at large spatial scales have generally focused on far smaller taxonomic groups (e.g., Riddle et al. 2000; vertebrates from Baja California, North America; Petit et al. 2003; trees from Europe; Kadereit et al. 2005, dicots from the Mediterranean basin).

We hypothesized that we would detect a west-east trend of increasing genetic diversity across all taxa, if the demographic (and thus genetic) clinal imprint left by the climate of the last glacial cycle on resident populations in refugia was stronger than the imprints left by the incongruent Holocene recolonization patterns of different species from different refugia (Taberlet et al. 1998). In contrast, if recolonization from disparate refugia across multiple taxa is the dominant signal for current patterns of *GDpop*, we would not expect to find such a longitudinal imprint across taxa. Refugia have been identified in many different parts of the region. For example, Médail and Diadema (2009) in their analysis of plant genetic patterns in the Mediterranean found that of 52 refugia identified, 33 were in the western Mediterranean and 19 in the eastern Mediterranean (non-significantly different from an equal distribution in each zone). At species level, we expected different trends depending on where refugia were located. We expected that this trend would be weaker for studies using *GDpop* measures giving higher weight to frequent alleles because rare alleles are more likely to disappear with recolonization and bottleneck events than do frequent alleles.

We also predicted that, depending on their position in the tree of life, the different taxa would respond differently to longitude. We expected that their responses would depend on:

- their life-history traits (low vs. high mobility);
- their bioclimatic requirements (particularly in plants, depending on their over-wintering abilities and their temperature requirements, and thus their sensitivity to local glacial climate); and
- their location within distribution areas (islands vs. continents and southern vs. northern Mediterranean, for which demographic effects and migration possibilities are different).

## Material and Methods

### Gathering data from published sources

**Figure 1:**
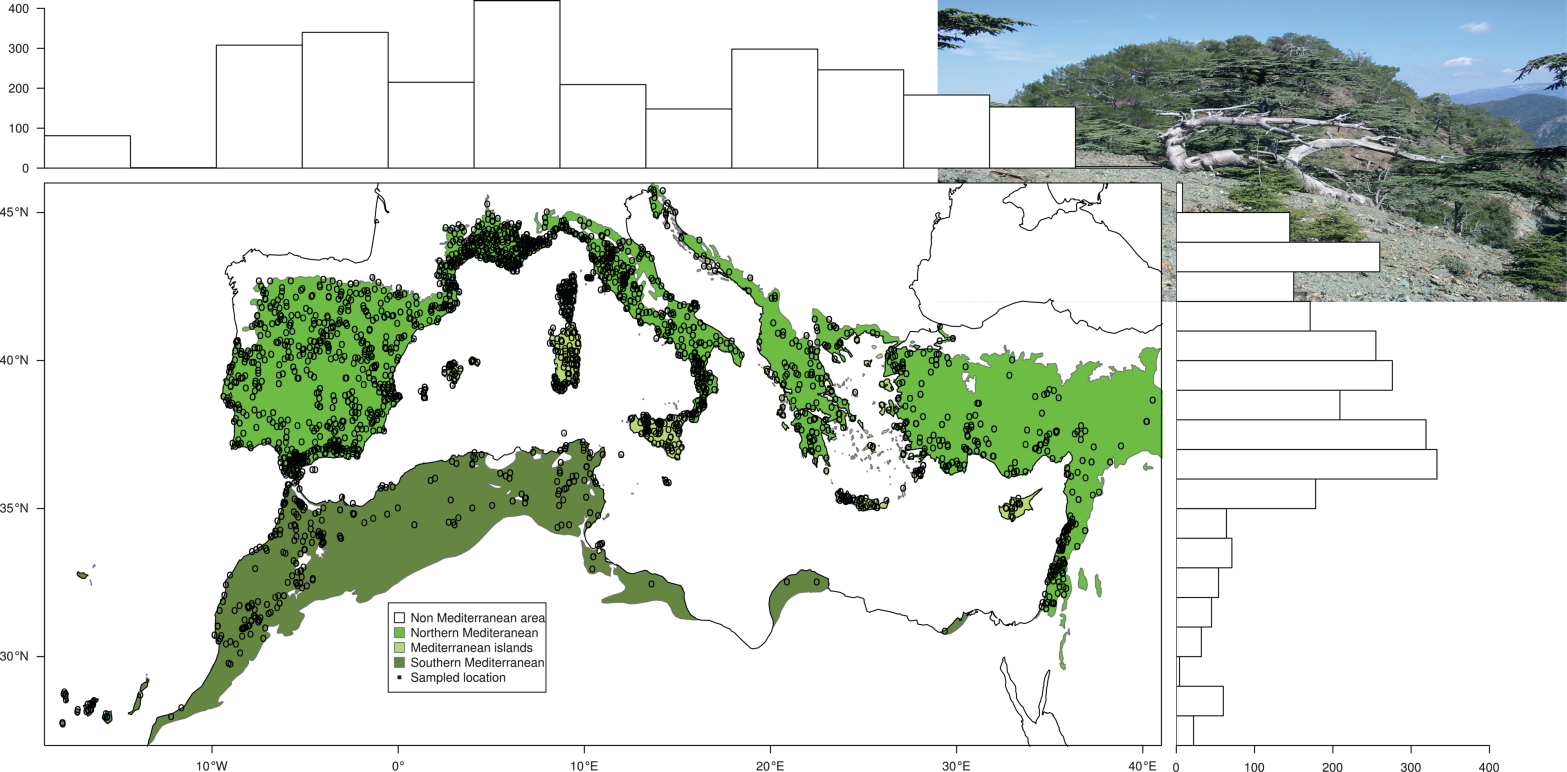
Map of the Mediterranean Basin showing the eco-climatic envelope as defined by Olson et al. (2001) and the geographic partition (North vs. South vs. islands) tested in our study. Each black circle represents a location sampled in the meta-analyzed raw studies. Histograms for latitudinal and longitudinal distributions of locations sampled in raw studies are given above and to the right of the map. The photographed tree in the upper right corner is a *Cedrus brevifolia* individual on a ridge in its natural habitat of the Trohodos mountains of Cyprus.

We collected published population genetic studies of terrestrial plant and animal species whose distributions were at least partially found within the Mediterranean basin, from endemic to widespread (Fig. 1). For this, we searched the Web of Knowledge for references published between January 1980 and October 2009. We used the following search expressions: “population genetics,” “phylogeo*,” “genetic diversit*,” “Mediterr*,” and individual Mediterranean country names. We also examined the references included in all retrieved publications for additional references.

### Defining the geographic zone of interest and population sample size

From these papers, we selected all populations with *GDpop* estimates that were included within the Mediterranean basin. We used the delineation of the Mediterranean basin defined by Olson et al. (2001), which is the standard currently used by the World Wildlife Fund (WWF) to define the world eco-regions. We used a geographic information system (GIS) for selecting among published studies, which population to allocate to that geographic envelope and to further qualify populations as continental versus insular and northern versus southern Mediterranean (Fig. 1).

### Constructing a database of *GDpop* estimates

We explain in details in Data S1 how we constructed our database. The list of published studies used is referenced in Data S2.

### Raw data analysis and calculation of effect-sizes

No published paper we retrieved had testing for a correlation between longitude and *GDpop* as its primary goal. We used the raw data from these studies to correlate the longitudinal position of each population with its *GDpop*. The statistics we used was the Pearson product–moment correlation coefficient.

The populations tested are not located on a strait longitudinal line, but rather span a small latitudinal gradient (which reaches its maximum in each of the Mediterranean peninsulas). Also, not all organisms remained in the close vicinity of their glacial refugia after Holocene warming (receding edge populations may have moved farther away from refugia than rear edge populations or endemic species, Jump et al. 2009). Thus, in order to account for the effect of latitude, we calculated a partial correlation coefficient, which measured the degree of association of *GDpop* with longitude while latitude was held constant. Partial correlation coefficients in meta-analysis represent the relationship between the independent and the dependent variable while controlling for other factors (Rosenthal and DiMatteo 2001; Keef and Roberts 2004). In our case, the controlling factor was always the same, latitude.

The partial correlation coefficients were transformed using Fisher's *Z*-transformation and used as effect-sizes: *Z =* 0.5 × ln(1 + *r*/1 − *r*), where *r* is the partial correlation coefficient. Finally, effect-sizes (*Z*-transformed partial correlation coefficients) were weighted by the inverse of their asymptotic variance (see Aloe and Becker 2009 for weighting partial coefficients in meta-analysis), which was calculated as *Vz* = 1/(*n*−3), where n is the number of sampled populations in the source study. Finally, summary-effects (*Zr*) were computed as the weighted mean of the individual effect-sizes using a fixed-effect meta-analysis model. We chose a fixed-effect model (Borenstein et al. 2009) because we were interested in testing primary factors affecting *GDpop* that acted in a similar way across all species and were expressed non-randomly across the whole Mediterranean basin.

As data in our primary dataset are not all independent (several papers in our dataset address the same species), we tested the effect of non-independence on Type I error rates as well as on the precision of summary-effects (Hartung et al. 2008) using several methods (see Data S3). As the redundancy of our raw data affected neither the direction of the relationship between *GDpop* and longitude nor its significance, we decided to use the entire dataset in the following meta-analyses and to not perform any statistical treatment to reduce redundancy.

Data processing, effect-size computations as well as sensitivity analyses were performed using R packages *plyr v0.1.9* (Wickham 2011), *MAc v1.1* (Del Re and Hoyt 2010) and custom scripts for sensitivity analyses (available upon request), under R v2.11 (http://www.r-project.org/), whereas the meta-analysis procedure was performed using MetaWin (Rosenberg et al. 2000).

### Exploring moderators of the summary-effects

#### Moderators related to primary study design

##### Effect of the sampling geographic range in the primary studies

If we hypothesize a general and homogeneous link of *GDpop* with longitude (supposing that longitude is a proxy for the same phenomenon for all species/studies), then we may expect that the wider the range of population sampling across the Mediterranean basin in the primary studies was, the greater the probability of detecting a positive mean effect-size *Zr* would be. Also, *Zr* might be affected by the position of the range within the Mediterranean basin. We tested these relationships by regressing each *Zr* and their corresponding longitudinal span calculated as the absolute value of the difference in degrees of longitude between the easternmost and the westernmost populations and each *Zr* and their corresponding mean longitudinal coordinate.

##### Choice of genetic marker and *GDpop* metric

The choice of the genetic marker may impact the sign of the correlation between *GDpop* and geography because they may reflect different processes acting at different spatial and time scales (ecological vs. evolutionary). They may also reflect different demographic histories via their different effective population size or sex-related transmission. Discrepancies have classically been found by phylogeographers between the nuclear and the mitochondrial DNA (Petit and Vendramin 2007). We thus tested marker-type effects by categorizing them depending on their inheritance type (male, female, or bi-parental inheritance), which may be related to an effect of dispersal ability. As foundation events or distance to refugia may be imprinted differently on the different types of *GDpop* measures (see the *Fagus sylvatica* example in Comps et al. 2001), we tested metric type effects by categorizing *GDpop* measures as either “equitability” or “richness” measures (see Introduction).

#### Biogeographic effect (north vs. south, continents vs. islands)

We tested biogeographic effects by categorizing the effect-sizes as either northern or southern Mediterranean, and as either from continents or islands.

#### Plant species biological attributes and ecological requirements

There may be a strong confounding effect between taxonomy and marker type in our general dataset. Specifically, cpDNA effects may be due to the type of DNA used or to traits specific to plants as this type of DNA is not present in animals. Thus, we used the part of our dataset restricted to plants to retest for marker-type effects on overall trends and also to test for the imprint of biological attributes and ecological requirements on *GDpop* in the Mediterranean.

Along with demographic processes, many life-history traits can be responsible more or less deterministically for spatial *GDpop* differences. For example, generation time is a life-history trait that will moderate the imprint of past demographic events on the measured *GDpop*. As a first general and very coarse proxy of those traits, we used taxonomy and we characterized all species by their family, class, and kingdom names.

For each plant species, we also recorded several biological and ecological attributes using information from Quézel and Médail (2003), Pignatti (1982), Rameau et al. (2008) as well as the Telabotanica web database (http://www.tela-botanica.org/ consulted in April 2009). We coded the altitudinal thermo-climatic belts (from thermo- to oro-Mediterranean, Quézel and Médail 2003) where each plant species was predominantly found. Bioclimatic requirements have been found to affect *GDpop* (Kadereit et al. 2005; Fady and Conord 2010; Soto et al. 2010). Species with higher temperature requirements may be more sensitive to demographic fluctuation in the Mediterranean as they will have been more strongly impacted by unfavorable cold climate during the cold cycles of the Quaternary. We also categorized each plant species according to their pollen and seed dispersal type (see Fig. 4d and e for the detailed types within categories). Seed dispersal type may influence *GDpop* as a result of migration, as illustrated by the comparison of beech versus hornbeam in Europe. Beech with its animal-dispersed nuts conserved more genetic diversity when crossing mountain barriers than did hornbeam with its winged seeds (Coart et al. 2005). Finally, each species in our database was categorized according to its Raunkiaer life-form, which is based on the position of the plant's buds during the unfavorable season, and therefore it may be a proxy of life-history traits playing an important role in the survival of the species under harsh conditions at the LGM.

## Results

### Overall effect-sizes and role of range, taxonomy, and DNA markers

**Figure 2:**
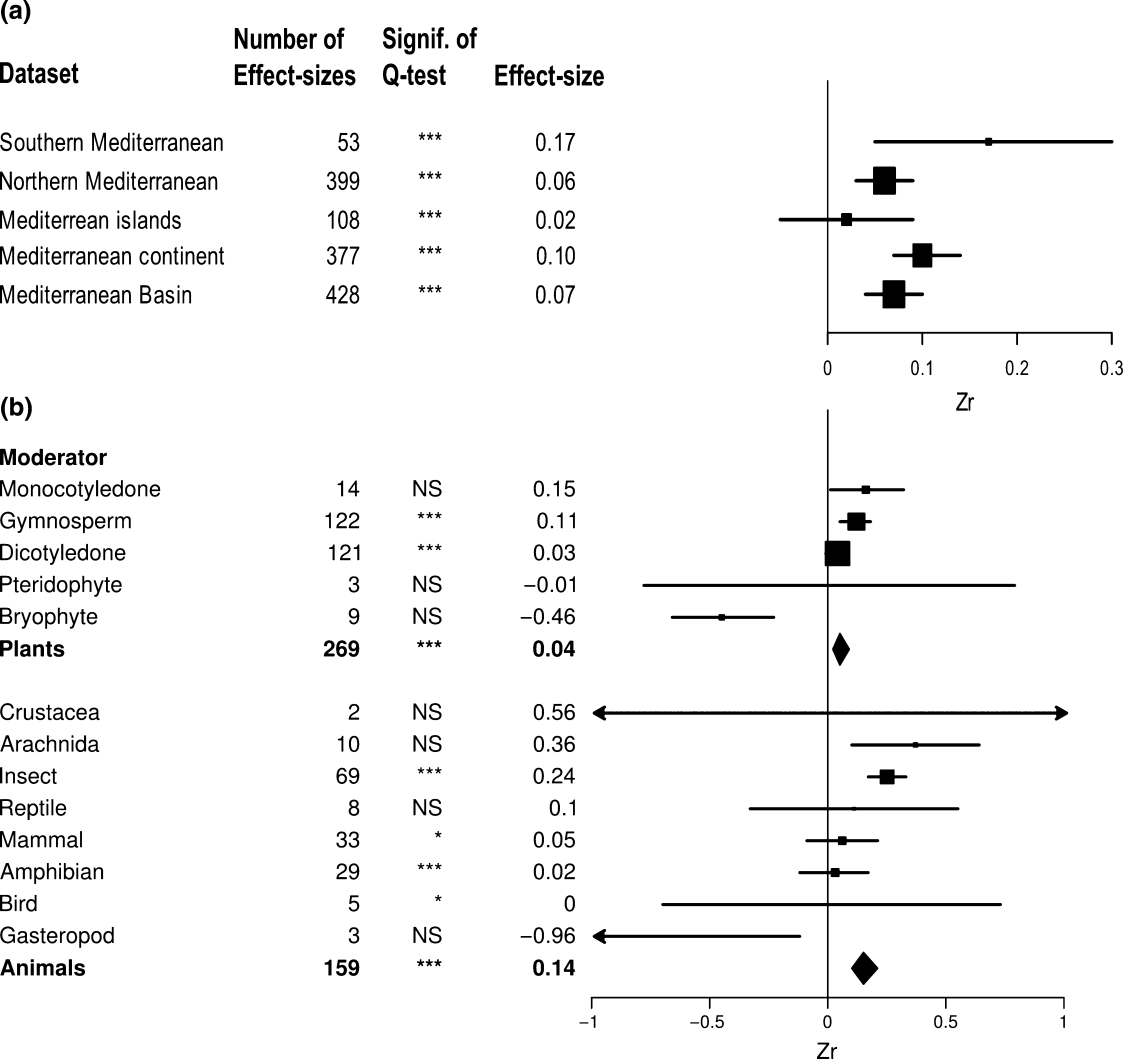
(a) Mean effect-sizes across the Mediterranean basin and for different biogeographic envelopes. Each square, whose size is proportional to the number of effect-sizes, indicates mean values and each bar the 95% confidence interval around the mean. Bars not intercepting the *Y*-axis indicate a significant correlation between *GDpop* and longitude. When the mean is on the positive side of the *X*-axis, this correlation is positive, indicating that *GDpop* increases from west to east. Bars with arrows indicate that the 95% confidence interval falls outside the limits of the figure. Diamonds highlight mean values for plants and animals. All Q-tests are highly significant (*P* < 0.0001), indicating that significant differences between effect-sizes remain within each biogeographic category. Nota bene: northern/southern and continent/island effect-sizes do not add up to the total number of effect-sizes in the Mediterranean basin because raw data studies may include species with populations present north and south of the Mediterranean and both on continents and on islands. (b) Mean effect-sizes across the Mediterranean basin for five plant and eight animal classes, and mean values for the plant and animal kingdoms. Classes are arranged in decreasing effect-size order. Square and error bars should be interpreted as indicated for (a).

Overall, there was a positive and significant correlation between *GDpop* and longitude in the Mediterranean (Fig. 2a). Within-population genetic diversity decreases from east to west in the Mediterranean (Table 1; Data S4). Of the 428 effect-sizes generated from 143 plant and animal species from 156 published studies in our meta-analysis, 54% showed a positive effect-size. Considerable heterogeneity was found (*Q* = 1180, df = 427, *P* < 0.0001) leading to the tests of the categorical moderators reported below. The general longitudinal trend in *GDpop* was affected by sampling range and range type, by marker and metric types, by taxonomy (phylogeny), by biological traits, and by ecological requirements.

**Table 1:** Description of the dataset and summary statistics: Geographic range (including islands) from Olson et al. (2001)

Taxonomic group	Species no.	Studies no.	Effect-sizes no.	mtDNA*	cpDNA*	cpDNA SSR*	nDNA*	nDNA Isozymes*	nDNA SSR*
Bryophyte	2	4	9	–	0	0	5	4	0
Dicotyledone	47	40	121	–	33	13	88	56	16
Gymnosperm	17	55	122	–	28	28	93	76	4
Monocotyledone	7	6	14	–	2	2	12	9	3
Pteridophyte	1	1	3	–	0	0	3	0	3
Total plants	74	106	269	–	63	43	201	145	26
Amphibian	14	7	29	6	–	–	23	23	0
Arachnida	6	4	10	0	–	–	10	10	0
Birds	3	3	5	5	–	–	0	0	0
Crustacea	1	1	2	2	–	–	0	0	0
Gasteropod	1	1	3	0	–	–	3	3	0
Insect	30	26	69	17	–	–	54	27	18
Mammal	10	11	33	8	–	–	25	12	12
Reptile	4	4	8	0	–	–	8	5	3
Total animals	69	57	159	36	–	–	123	80	33
Total	143	163	428	36	63	43	324	225	59

* Number of effect-sizes.

#### Sampling range and range type

Both sampling range span and the mean longitudinal position of the studies were significantly but weakly correlated with *Zr* (*r* = 0.0043, *P* < 0.0001 and *r* = 0.0086, *P* < 0.0001, respectively). Widely distributed species and species from the eastern part of the Mediterranean tended to have more significantly positive *Zr* than others. The *Zr* was five times higher for continents than for islands and almost three times higher for the southern than for the northern Mediterranean (Fig. 2a). However, *Zr* was positive and significant for all geographic envelopes except for Mediterranean islands. All tests based on Q statistics rejected the null hypothesis of homogeneity among effect-sizes within category, that is, that all studies shared a common effect-size (*P* < 0.0001, Data S4). This suggested that there was more variability among the effect-sizes of a category than expected by chance and justified the search and testing of moderating variables.

Studies in our dataset sampled a majority of northern Mediterranean populations (399 effect-sizes in the north compared with 53 for the south). Differences in *Zr* between the northern and southern Mediterranean were not biased by high order taxonomic differences. The number of effect-sizes belonging to the six different phyla of the database (Arthropods, Mollusks, Chordata, Bryophytes, Pteridophytes, Spermaphytes) were not significantly different between the northern and southern Mediterranean (contingency χ^2^ test = 12.05, df = 5, *P* = 0.06), although they were between continents and islands (contingency χ^2^ test = 15.448, df = 5, *P* = 0.01).

#### Phylogeny and taxonomic group

**Figure 3:**
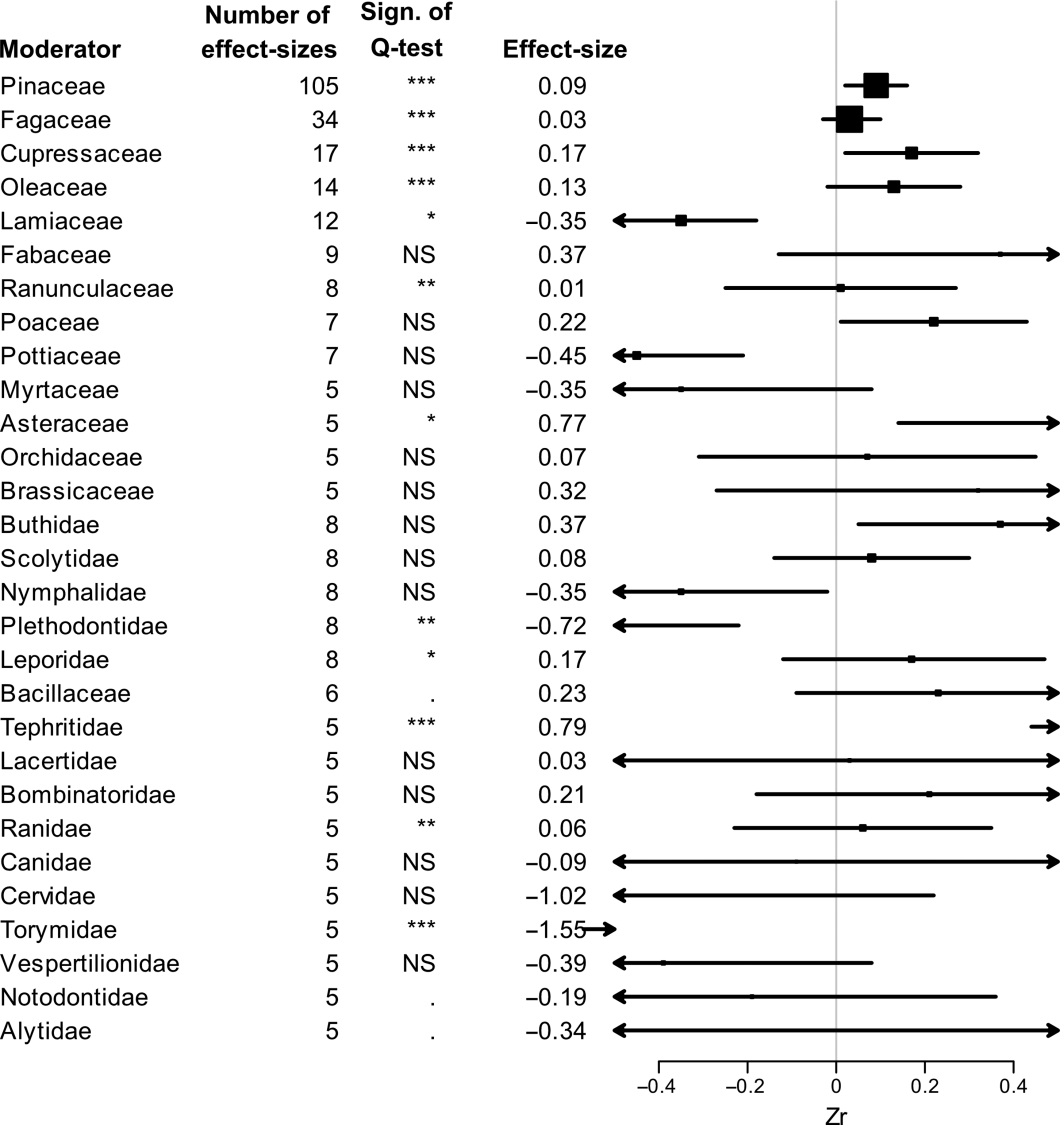
Mean effect-sizes across the Mediterranean basin for 12 plant and 17 animal families, arranged in decreasing effect-size number per category. Of 62 possible families represented in the dataset, only the 29 with a number of effect-sizes over 5 are represented here. Square and error bars should be interpreted as indicated for Fig. 2a.

The *Zr* computed for the animal and plant kingdoms were both positive and significant. *Zr* for animals was more than three times higher than that of plants. The two *Q*-tests rejected homogeneity of *Zr* within plants and animals (Fig. 2b), indicating that within each kingdom, finer level groups departed significantly from the positive trend. At a finer taxonomic level, five classes out of 13 had a summary-effect not intercepting the Y-axis (Bryophytes, Gymnosperms, Arachnida, Insects and Gastropods, Fig. 2b). Heterogeneity tests were non-significant for all class levels with less than 10 effect-sizes except for birds (Fig. 2b), suggesting consistent responses among the members of these groups; however, the *Q*-test is not very powerful and may fail to detect true heterogeneity, particularly in such small groups. The remaining groups were highly significantly heterogeneous. At the yet finer taxonomic level of the family, significant longitudinal *GDpop* structures could be observed in the Pinaceae, Cupressaceae, Poaceae, and Asteraceae (positive summary-effects) and in the Lamiaceae, Nymphalidae, and Pottiaceae (negative summary-effects, Fig. 3). As the taxonomic sampling was unbalanced, we ran the meta-analysis excluding successively the most represented groups, from the higher taxonomic level to the finer. Excluding the Phanerophytes (192 effect-sizes) had no effect on the *Zr* computed for the whole dataset and for the continental Mediterranean. However, *Zr* for the northern Mediterranean became non-significant, whereas *Zr* for southern Mediterranean and for islands increased and even became significant for islands. Then, excluding gymnosperms (122 effect-sizes) confirmed the patterns observed when excluding phanerophytes except for the southern Mediterranean *Zr*, which became non-significant. In animals, two families showed a positive trend (Buthidae and Tephritidae), whereas two others showed the opposite trend (Plethodontidae and Torymidae) (Fig. 3).

#### Markers type and metric type

**Figure 4:**
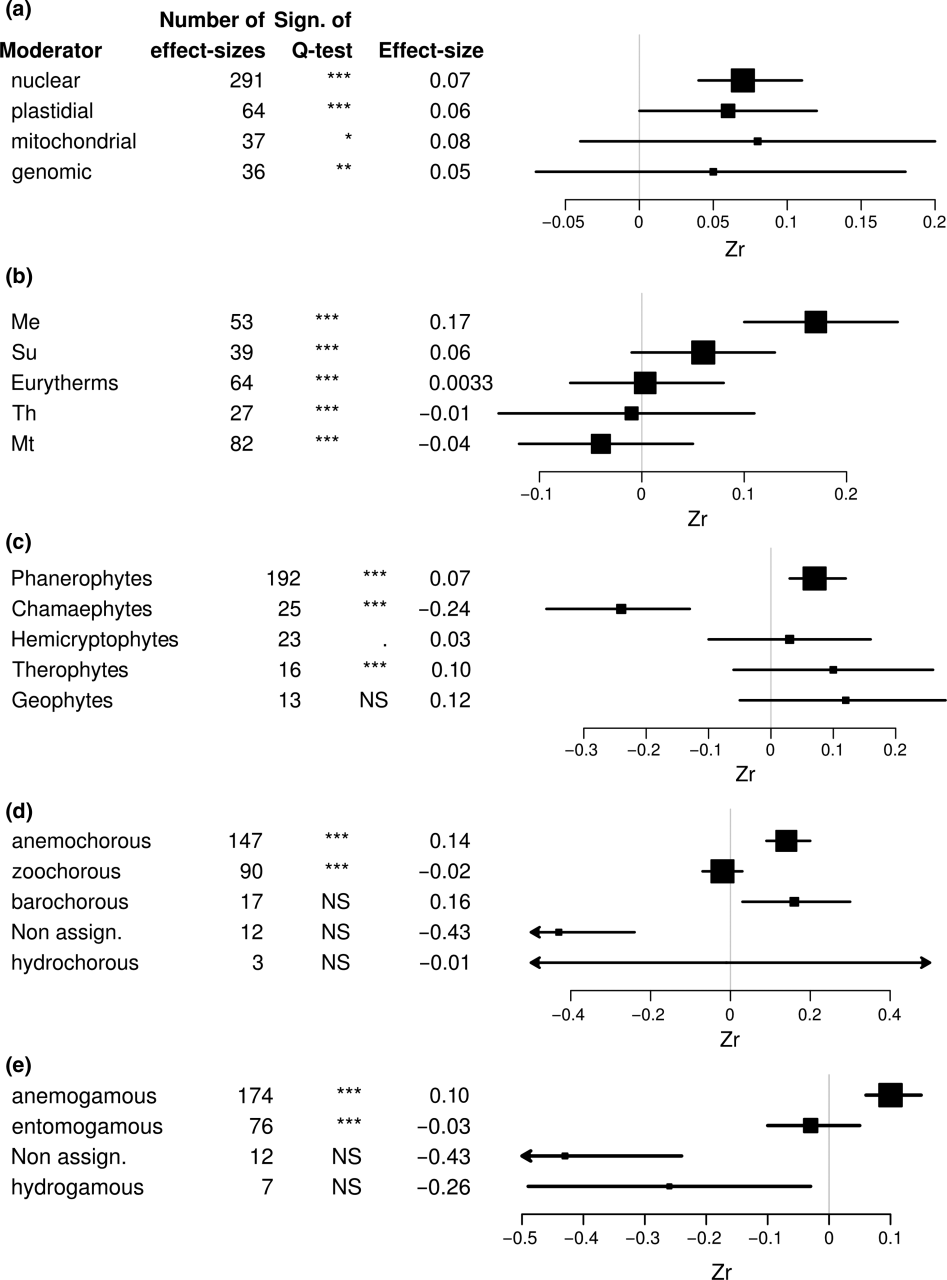
Square and error bars should be interpreted as indicated for Fig. 2a. (a) Mean effect-sizes across the Mediterranean basin for marker type. The category “genomic” refers to unassigned marker types. (b) Mean effect-sizes (ordered from most positive to most negative) of Mediterranean basin plants for ecological requirements. The categories refer to altitudinal belts where plant species are predominantly found: “*Me*” is meso-Mediterranean, “*Su*” is supra-Mediterranean, “*Eurytherms*” refers to plant species found across several altitudinal belts, “*Th*” is thermo-Mediterranean and “*Mt*” is mountain-Mediterranean (see Quézel and Médail 2003). Square and error bars should be interpreted as indicated for Fig. 2a. Nota bene: effect-sizes for ecological requirements do not add up to the total number of effect-sizes in plants because raw data communicated by some authors were pooled at the genus level or because data included species for which we were not able to retrieve their ecological requirement. (c) Mean plant effect-sizes (arranged in decreasing effect-size frequency per category) across the Mediterranean basin for Raunkiaer biological types. Phanerophytes are woody plants with over-wintering buds situated over 50 cm from the ground, chamaephytes are low-growing perennials (often woody plants) with wintering buds below 50 cm in height, hemicryptophytes are (often 2-year cycle) perennials with ground-level wintering buds, geophytes are plants with bulbs or rhizomes (wintering buds below ground level), and therophytes are annuals (wintering organs as seeds). Nota bene: Bryophytes were not assigned a Raunkiaer type (nine effect-sizes). (d) Mean plant effect-sizes (arranged in decreasing effect-size frequency per category) across the Mediterranean basin for seed dispersal types. Anemochorous plants have wind-dispersed seeds, zoochorous plants animal-dispersed seeds, barochorous plants gravity-dispersed seeds, and hydrochorous plants water-dispersed seeds. (e) Mean plant effect-sizes (arranged in decreasing effect-size frequency per category) across the Mediterranean basin for pollen dispersal types. Anemogamous plants have wind-dispersed pollen, entomogamous plants insect-dispersed pollen, and hydrogamous plants water-dispersed pollen.

Nuclear and organelle markers showed a positive *Zr* of the same order of magnitude, although mitochondrial *Zr* was not significantly different from zero (Fig. 4a). When assessing the effect of the inheritance of the genetic marker, we found that bi-parentally and paternally inherited markers yielded a significant *Zr*. On the contrary, *Zr* for maternally inherited markers (mitochondrial DNA in all species of our dataset and plastidial DNA in angiosperms) was positive but non-significant (not shown), indicating that *GDpop* for maternally inherited markers does not significantly increase with longitude. *Zr* for both “equitability” and “richness” indices were significant, positive, and similar in magnitude. Excluding island populations from the dataset increased *Zr* values. *Zr* for “equitability” was three times higher when restricting the dataset to the southern Mediterranean envelope, whereas *Zr* for “richness” stayed constant, but became non-significant (not shown).

### Effect-sizes in plants and role of ecological requirements and biological traits

The summary-effects for bi-parentally and maternally inherited markers decreased and became (or remained in the case of maternally inherited markers) non-significant after excluding animals from the dataset (not shown). In plants, positive and significant effects-sizes were thus found for plastidial DNA and paternally inherited DNA (gymnosperm plastidial DNA).

#### Ecological traits

We were able to assign 201 effect-sizes of plant species to a bioclimatic belt without ambiguity (Fig. 4b). The remaining species being found in two or more belts were labeled as “eurytherm” species (64 effect-sizes). The true Mediterranean ecological group (meso-Mediterranean) had the highest positive *Zr* and was the only significant group. The supra-Mediterranean group had a positive but non-significant *Zr*. The category with the highest sample size (mountain-Mediterranean) had a negative and non-significant *Zr*, and the gymnosperms contributed predominantly to this group (70 of the 82 effect-sizes). The group with the highest requirements in terms of temperature (thermo-Mediterranean) had an almost null *Zr*. Heterogeneity was significant in all groups.

#### Biological traits

Phanerophytes (trees and shrubs) represented approximately 2/3 of the plants in our dataset and showed a positive and significant *Zr* (Fig. 4c). Among them, gymnosperms had a positive *Zr* (see above). Dicots among phanerophytes showed a contrasting pattern depending on the geographic envelope: at Mediterranean basin level, the effect was positive but non-significant; whereas it was strongly positive for the continental group (0.10 [0.04; 0.16] 95% CI, *N* = 65) and strongly negative for islands (−0.56 [−0.71; −0.40] 95% CI, *N* = 17). Contrasting with the other moderator analyses, the southern group yielded a negative trend while the northern group showed a positive one. Chamaephytes (low-growing perennials with over-wintering buds below 50 cm) were the only group showing a negative (although non-significant) *Zr*.

Seed dispersal mode affected *Zr* values: Anemochorous (wind-dispersed, contributing to more than half of the effect-sizes) and barochorous (gravity-dispersed) plants showed a significant and positive *Zr*, whereas zoochorous (animal-dispersed) plants had a non-significant *Zr* (Fig. 4d). Pollen dispersal type also affected *Zr* values. Although calculated from a small sample size, the hydrogamous (water-dispersed pollen) plants showed a homogeneous negative trend (Fig. 4e), whereas entomogamous (insect-dispersed pollen) species had a non-significant *Zr* and anemogamous (wind-dispersed pollen) species had a positive and significant *Zr*.

## Discussion

Organization of genetic diversity in Europe mostly follows latitudinal routes of recolonization dating from the Holocene (Petit et al. 2003). In the Mediterranean, although a latitudinal imprint exists, our analysis demonstrates the existence of a longitudinal imprint on genetic diversity. Using a meta-analysis on 143 plant and animal species, we found that overall within-population genetic diversity of plants and animals increases significantly from west to east in the Mediterranean basin, both in southern Europe and in North Africa, and for continental, but not for island species. The longitudinal trend was not found in all taxonomic groups, however. This result broadens the evidence provided by Fady and Conord (2010) beyond tree species to include such taxonomic groups as arthropods, well represented in the dataset, gymnosperms, and monocots. Other well-represented groups including dicots, mammals, and amphibians do not follow the trend. Several poorly represented groups such as gastropods and bryophytes demonstrate a significant opposite trend, or no trend (reptiles and ferns).

### What are the processes that shaped current genetic diversity longitudinally across the Mediterranean basin?

Trying to detect events/processes responsible for longitudinal imprints on genetic diversity in the Mediterranean may be challenging. The effects of older events such as divergence/diversification linked to vicariance during the Pliocene (Blondel and Aronson 1999; emergence of geographic barriers) may coincide with that of younger events, such as demographic bottlenecks during the Last Glacial Maximum, Holocene colonization events, admixture from secondary contact (Petit et al. 2003) or hybridization with closely related species (Papageorgiou et al. 2008).

However, it can be assumed that many Mediterranean species and populations have remained closer to their glacial refugia than their European counterparts and thus carry imprints (however attenuated) that commonly affected glacial refugia. A meta-analysis, using a fixed-effect model, precisely makes this assumption (Borenstein et al. 2009). It thus assumes that a common set of drivers affected *GDpop* across species along a longitudinal gradient in the Mediterranean basin. By analyzing the effect of moderators on summary-effect sizes, our goal was to discuss the most parsimonious explanations for such a trend.

### Effect of past climate on resident populations or effect of recolonization on genetic diversity in the Mediterranean

#### Biological and life-history traits

We show that both plants and animals display a trend of increasing *GDpop* from west to east in the Mediterranean. We expected that, on average, the trend would be due more to migration effects (recolonization patterns) in mobile or high gene flow organisms, and more to local climatic effects in sessile or low gene flow organisms. With perhaps a difference in intensity between the two trends, that due to migration being less pronounced because of high gene flow and recurrent genetic exchange between populations blurring post recolonization foundation effects. Our data show that reality may be more complex because both highly mobile (insects) and more sessile (arachnids) animals showed trends similar in direction and magnitude. In arachnids, a group only comprising scorpions from the southern part of the Mediterranean basin in our dataset, the effect could be related to local past-climate effects. The pattern shown by insects could reveal a link between their contemporary genetic structure and the mirrored structure of the plants they exploit. Recent studies have indeed illustrated the link between levels of diversity in keystone organisms such as trees and in their phytophagous-associated organisms (Crutsinger et al. 2006; Whitham et al. 2006). The relationship has also been shown to hold true from local to region-wide scales (Bangert et al. 2006). Our study is certainly the first for trees and insects, indicating a clear common pattern of congruent *GDpop* at continental scale.

The absence of significant trend in mobile vertebrate groups such as birds, amphibians, and mammals could reflect the coexistence of multiple Holocene recolonization routes from multiple refugia among species. For example, in two species of rodents from the genus *Apodemus*, LGM survival had two very different outcomes, with *A. flavicollis* disappearing from the Iberian Peninsula, whereas *A. sylvaticus* survived only there. The subsequent Holocene recolonization of Europe by these two currently sympatric species left two diverging imprints on genetic diversity (Michaux et al. 2005). In yet another species of rodent, the shrew *Crocidura russula*, refugial populations were located in North Africa from which western European Holocene populations derive (Cosson et al. 2005). As for the bank vole, *Clethrionomys glareolus*, most of its Holocene European range was recolonized from central European refugia although Mediterranean refugia existed (Deffontaine et al. 2005). In this group of mammals, the exception may be the rule in terms of LGM survival and Holocene recolonization, which is indicated by a non-significant trend of genetic diversity in our meta-analysis.

The only plant type (sensu Raunkiaer) displaying a negative trend in *GDpop* was the chameaphytes. Because of their ground-level overwintering buds, chamaephytes are better suited to resist cold snowy winters than dry winters (Taulavuori et al. 2011). Snow may in fact be beneficial to their overwintering, thus potentially keeping larger populations during the LGM in the western than in the eastern Mediterranean.

The positive trend found in phanerophytes (i.e., woody plants having buds at least 50 cm above the ground) overlaps with that of wind-dispersed species and may reflect both local demographic effects under LGM climate and long distance Holocene recolonization. Seed dispersal by gravity, however, does not allow for rapid and long-range dispersal. Thus, the positive trend found in gravity-dispersed plant species matches the expectation of an effect of local *LGM* climate on genetic diversity. Conversely, animal-dispersed species depend on their dispersers' behavior for survival (Scofield et al. 2010). Their lack of trend in genetic diversity may mirror the diversity of vertebrate Holocene recolonization routes highlighted above for mammals.

#### Ecological requirements in plant taxa

When assessing the impact of ecological (temperature) requirement on plants, we expected to find an increasing positive effect on *GDpop* from low- to high-elevation (from less to more cold tolerant) plant species. The rationale for this expectation was that the unfavorably cold *LGM* climate should affect more strongly population size in species with higher temperature requirements as they became trapped in reduced-size habitats compared with those of lower temperature requirement species. In contrast, species adapted to colder climates such as supra- and mountain-Mediterranean species for example, benefiting from larger habitats during the LGM, should not have suffered demographic bottlenecks as did the more truly Mediterranean group. Thus, a west to east oriented clinal climate at the LGM should result in a stronger west to east clinal *GDpop* structure in true Mediterranean species (“*Me*” in Fig. 4b) than for species with other ecological requirements. The strength of the effects followed our expectations, with true Mediterranean plants showing a stronger cline of west-east increasing *GDpop* than Supra- and Mountain-Mediterranean plants (“*Su*” and “*Mt*” in Fig. 4b). The group of plants with no precise thermal requirement showed no significant cline. The non-significant slightly negative summary-effect of the “*Th*” group was more surprising. We expected this group of warm climate species to have been impacted even more strongly than other categories by cold climate at the *LGM* whether through strong bottlenecks or extirpation and subsequent migration/recolonization. Several explanations are possible. “*Th*” species may have survived locally without stronger loss in *GDpop* in the west as compared with the east, although we have no evidence to support this explanation. “*Th*” species could also have recolonized from glacial refugia not situated in the eastern Mediterranean, which was demonstrated for *Cistus ladanifer* (which came back into Spain from North Africa via the Strait of Gibraltar) by Guzmán and Vargas (2009). Finally, the thermo-Mediterranean belt is known to have endured severe human impact throughout the millennia, possibly obscuring clinal climatic and recolonization effects on *GDpop*. Interestingly, the two species generating most of the effect-sizes in this group were species strongly impacted by humans (*Pinus pinea*, *Olea europaea*), both being valuable food crop.

#### Marker and metric types

Studies using maternally inherited markers are most of the time designed to detect phylogeographic signals and capture differentiation effects, for example, those due to the imprints of contraction and recolonization to and from Pleistocene refugia. Studies using paternally inherited (plastidial DNA in gymnosperms) or bi-parentally inherited markers are more often designed to detect local demographic signals (e.g., for conservation planning) resulting from current environmental drivers, in addition to phylogeographic signals. This confirms that the overall trend detected in our study is a within-population demographic bottleneck effect, more weakly detected in maternally than paternally and bi-parentally inherited genomes. Phylogeographic studies have shown that the two different metric types we have used here, richness and equitability, can be negatively correlated along the distribution range of species (Comps et al. 2001; Petit et al. 2003). In Europe, from refugia to the newly colonized areas, heterozygosity increases (merging of recolonization routes that originated from different refugia), whereas allelic richness decreases (founder effects along the recolonization route) with local diversity peaks in suture zones. We found no such significant difference in our analysis. The two metric types had congruent positive and significant summary-effects. Taken together, the global positive effects we measured for marker and metric types may indicate a stronger role of local climate over recolonization in shaping the genetic diversity of Mediterranean populations.

#### Biogeographic effects: south versus north and continents versus islands

The Mediterranean basin has a highly heterogeneous and fragmented geography (Blondel and Aronson 1999). Its different geographic compartments have likely experienced different past ecological conditions and evolutionary histories. The northern Mediterranean flora and fauna contain predominantly Nordic, Asian, and local elements, whereas the southern Mediterranean is predominantly made of Tropical and local elements (Quézel and Médail 2003). Because of its peninsulas, migration may have been more restricted in the northern Mediterranean than in the southern Mediterranean. Also, one might expect stronger demographic bottlenecks and stronger scale and size effects on islands than on the continent. Although the number of populations originating from the southern Mediterranean was eight times lower than in the north, its summary-effect was more strongly positive than that of northern Mediterranean populations. Also, in the southern Mediterranean, the equitability metric type was more strongly positive than the richness metric type (which was actually non-significant). Although the gradient was more restricted in its longitudinal span (mostly but not entirely limited to populations of the Maghreb) in the southern than in the northern Mediterranean, these results suggest that factors linked to local *LGM* climate may have more strongly affected genetic diversity in the Southern Mediterranean than those linked to recolonization.

The weakly positive summary-effect of Mediterranean islands is in sharp contrast with that of continents. It may reflect a Mediterranean “insularity syndrome” globally independent of climatic factors and more likely linked to high endemism (Médail and Diadema 2009) and/or early human impact (Vigne et al. 2009).

## Conclusions

Our population genetic diversity dataset covered an extensive range of animal and plant species. It also had the advantage of gathering data from species with strong economic importance (e.g., fruit trees and medicinal plants), strong ecological importance (e.g., forest trees) as well as endangered flagship species (e.g., butterflies and endemic plants). The taxonomic groups that were the most heavily sampled (gymnosperms for plants and arthropods for animals) showed a congruently positive summary-effect, that is, an increasing genetic diversity from west to east. These abundant taxonomic groups are thus good models for detecting trends and patterns affecting biodiversity in general. The propensity of the genetic diversity of trees to be an excellent testimony of the imprint of ancient evolutionary and demographic processes (given their long generation time) has been mentioned for some time (Petit et al. 2003; Petit and Hampe 2006) and it seems that arthropods can be added to this category.

Longitude is a strong structural element of biodiversity at gene level in the Mediterranean, both in southern Europe and in North Africa. Continental/oceanic longitudinal-type gradients also exist in many parts of the world. The generally overlooked role of longitude in shaping species ranges and genetic diversity deserves stronger focus (Stewart et al. 2010). Taken together, our results suggest that, on top of a genetic structure inherited from the existence of glacial refugia (which phylogeography is increasingly demonstrating as being very complex, Leppanen et al. 2011), local climate during the *LGM* durably affected the demography of resident populations in the Mediterranean, observable as a weak but highly significant longitudinal cline of genetic diversity.

For conserving and sustainably managing biodiversity, global or region-wide assessments are needed beyond the idiosyncrasy of single species or single taxonomic groups to detect trends and large-scale patterns. Meta-analyses, by making it possible to compare already available data acquired within unrelated studies, provide an interesting framework for these assessments. Already successfully used in ecology to test theoretical predictions (e.g., Rapoport's law predicting an increase in species range with latitude, Ruggiero and Werenkraut 2007), we have shown that meta-analyses can also be powerful to test the determinants of large-scale biodiversity patterns.

Finally, our findings can now be compared with other measurements of past, current, and expected biodiversity (e.g., species and functional traits) and their congruence tested (Devictor et al. 2010), provided that appropriate databases (species, ecosystems, past and present climate) exist or can be constructed at relevant scales. Data available for estimating biodiversity at gene level remain critically insufficient in the Mediterranean. For example, the Mediterranean comprises approximately 200 mammal species and more than 300 bird species (Blondel and Aronson 1999), whereas our dataset only included 10 mammal and three bird species! This remains a major challenge in the poorly politically structured Mediterranean, but also in other regions of the world where biodiversity is high and rapidly declining.

## Supporting Information

**Data S1.** Constructing a database of *GDpop* estimates.

**Data S2.** List of the 156 published studies used in the meta-analysis for correlating GDpop with longitude in the Mediterranean basin.

**Data S3.** Testing for biases related to non-independence of data.

**Data S4.** Effect size values used in figures 2 to 4.

**DataS5.** List of taxa found in primary studies with their reference code (see Data S2) and taxonomical levels used as categorical moderators in the meta-analysis.
