# Supplementary material for: Large-scale longitudinal gradients of genetic diversity: a meta-analysis across six phyla in the Mediterranean basin
Source: Ecol Evol. 2012 Sep 14;2(10):2600–14. doi: 10.1002/ece3.350 (PMC3492785; doi:10.1002/ece3.350)
Supplement: Supplementary file 1 [file ece30002-2600-SD1.rtf]

Supplementary material I
Constructing a database of GDpop estimates:

We created a database where each line corresponded to an individual population from the Mediterranean, characterized by its geographical coordinates and GDpop estimates. Studies sometimes contained more than one species and a single species was sometimes studied by several published papers. Also, there was often more than one marker and/or GDpop measure used for each species and population within study. We recorded all measures for each population independently and generated a code for that redundancy within the study. When a primary study reported genotypic data from single individuals in sampled localities but with an extensive sampling effort across an area or across the whole Mediterranean Basin (as, for example, in Ronfort et al. 2006), we grouped neighboring localities into small regions for which we computed genetic diversity indices. The new georeferenced code for each of these artificial populations was the geometric center of all coordinates within the small region. For quality, we only kept in our database those studies for which sample size was more than 3 populations per study.  

Reference:
Ronfort J, Bataillon T, Santoni S, Delalande M, David JL, Prosperi JM (2006) Microsatellite diversity and broad scale geographic structure in a model legume: building a set of nested core collection for studying naturally occurring variation in Medicago truncatula. BMC Plant Biology, 6, 1-13.
