# Supplementary material for: Large-scale longitudinal gradients of genetic diversity: a meta-analysis across six phyla in the Mediterranean basin
Source: Ecol Evol. 2012 Sep 14;2(10):2600–14. doi: 10.1002/ece3.350 (PMC3492785; doi:10.1002/ece3.350)
Supplement: Supplementary file 2 [file ece30002-2600-SD2.rtf]

Supplementary material II. 
List of the 156 published studies used in the meta-analysis for correlating GDpop with longitude in the Mediterranean basin.   

Abellán, P.; Millán, A. & Ribera, I. (2009), 'Parallel habitat-driven differences in the phylogeographical structure of two independent lineages of Mediterranean saline water beetles', Molecular Ecology 18(9999), 3885-3902.

Affre, L.; Thompson, J. & Debussche, M. (1997), 'Genetic structure of continental and island populations of the Mediterranean endemic Cyclamen balearicum (Primulaceae)', American Journal of Botany 84(4), 437-451.

Afzal-Rafii, Z. & Dodd, R. S. (2007), 'Chloroplast DNA supports a hypothesis of glacial refugia over postglacial recolonization in disjunct populations of black pine (Pinus nigra) in western Europe', Molecular Ecology 16, 723-736.

Agrimonti, C.; Bianchi, R.; Bianchi, A.; Ballero, M.; Poli, F. & Marmiroli, N. (2007), 'Understanding biological conservation strategies: a molecular genetic approach to the case of myrtle (Myrtus communis L.) in two Italian regions: Sardinia and Calabria', Conservation Genetics 8, 385-396.

Agundez, D.; Degen, B.; von Wuehlisch, G. & Alia, R (1999), 'Multilocus Analysis of Pinus halepensis Mill. from Spain: Genetic Diversity and Clinal Variation', Silvae Genetica, 48, 3-4.

Ainouche, M. L.; Bayer, R. J.; Gourret, J. P.; Defontaine, A. & Misset, M. T. (1999), 'The allotetraploid invasive weed Bromus hordeaceus L. (Poaceae): Genetic diversity, origin and molecular evolution', Folia Geobotanica 34(4), 405-419.

Albaladejo, R. G. & Aparicio, A. (2007), 'Population Genetic Structure and Hybridization Patterns in the Mediterranean Endemics Phlomis lychnitis and P. crinita (Lamiaceae)', Annals of Botany 100, 735-746.

Alexandrino, J.; Froufe, E.; Arntzen, J. W. & Ferrand, N. (2000), 'Genetic subdivision, glacial refugia and postglacial recolonization in the golden-striped salamander, Chioglossa lusitanica (Amphibia: Urodela)', Molecular Ecology 9, 771-781.

Allegrucci, G.; Minasi, M. G. & Sbordoni, V. (1997), 'Patterns of gene flow and genetic structure in cave-dwelling crickets of the Tuscan endemic, Dolichopoda schiavazzii (Orthoptera, Rhaphidophoridae)', Heredity 78(6), 665-673.

Augustinos, A. A.; Mamuris, Z.; Stratikopoulos, E. E.; D'Amelio, S.; Zacharopoulou, A. & Mathiopoulos, K. D. (2005), 'Microsatellite analysis of olive fly populations in the Mediterranean indicates a westward expansion of the species', Genetica 125, 231-241.

Bagnoli, F.; Vendramin, G. G.; Buonamici, A.; Doulis, A. G.; González-Martínez, S. C.; Porta, N. L.; Magri, D.; Raddi, P.; Sebastiani, F. & Fineschi, S. (2009), 'Is Cupressus sempervirens native in Italy? An answer from genetic and palaeobotanical data', Molecular Ecology 18(10), 2276-2286.

Balfourier, F.; Charmet, G. & Ravel, C. (1998), 'Genetic differentiation within and between natural populations of perennial and annual ryegrass (Lolium perenne and L. rigidum)', Heredity 81, 100-110.

Bechir, A.; El Mousadik, A. & Pichot, C. (2004), 'Allozyme diversity of natural and introduced cypress stands in Morocco: consequences for management of genetic resources', Annals of Forest Science 61(7), 669-676.

Belaj, A.; Munoz-Diez, C.; Baldoni, L.; Porceddu, A.; Barranco, D. & Satovic, Z. (2007), 'Genetic diversity and population structure of wild Olives from the North-western Mediterranean assessed by SSR markers', Annals of Botany 100, 449-458.

Ben Othmen, A.; Chatti, N.; Ben Ali-Haouas, Z.; Ouldbrahim, I. & Said, K. (2004), 'Allozymic differentiation of Tunisian populations of Androctonus species and Buthus occitanus (Scorpiones : Buthidae)', Biological Journal of the Linnean Society 81(2), 255-265.

Boato, A. (1988), 'Microevolution in Solatopupa landsnails (Pulmonata Chondrinidae): genetic diversity and founder effects', Biological Journal of the Linnean Society 34(4), 327-348.

Boratynski, A.; Lewandowski, A.; Boratynska, K.; Montserrat, J. & Romo, A. (2009), 'High level of genetic differentiation of Juniperus phoenicea (Cupressaceae) in the Mediterranean region: geographic implications', Plant Systematics and Evolution 277(3), 163-172.

Boscherini, G.; Morgante, M.; Rossi, P. & Vendramin, G. G. (1994) Allozyme and chloroplast DNA variation in Italian and Greek populations of Pinus leucodermis. Heredity, 73, 284-290 

Bou Dagher-Kharrat, M.; Mariette, S.; Lefèvre, F.; Fady, B.; Grenier-de March, G.; Plomoin, C. & Savouré, A. (2007), 'Geographical diversity and genetic relationships among Cedrus species estimated by AFLP', Tree Genetics & Genomes 3, 1614-2942.

Breton, C.; Tersac, M. & Berville, A. (2006), 'Genetic diversity and gene flow between the wild olive (oleaster, Olea europaea L.) and the olive: several Plio-Pleistocene refuge zones in the Mediterranean basin suggested by simple sequence repeats analysis', Journal of Biogeography 33(11), 1916-1928.

Briese, D. T.; Espiau, C. & Pouchot-Lermans, A. (1996), 'Micro-evolution in the weevil genus Larinus: the formation of host biotypes and speciation', Molecular Ecology 5, 531-545.

Brito, P. (2005), 'The influence of Pleistocene glacial refugia on tawny owl genetic diversity and phylogeography in western Europe', Molecular Ecology 14, 3077-3094.

Bruna, S.; Portis, E.; Cervelli, C.; De Benedetti, L.; Schiva, T. & Mercuri, A. (2007), 'AFLP-based genetic relationships in the Mediterranean myrtle (Myrtus communis L.)', Scientia Horticulturae 113, 370-375.

Bucci, G.; Vendramin, G.; Lelli, L. & Vicario, F. (1997) Assessing the genetic divergence of Pinus leucodermis Ant. endangered populations: use of molecular markers for conservation purposes Theoretical and Applied Genetics, 95, 1138-1146 

Bucci, G.; Anzidel, M.; Madaghiele, A. & Vendramin, G. G. (1998), 'Detection of haplotypic variation and natural hybridization in halepensis-complex pine species using chloroplast simple sequence repeat (SSR) markers', Molecular Ecology 7, 1633-1643.

Bucci, G.; Gonzalez-Martinez, S. C.; Le Provost, G.; Plomion, C.; Ribeiro, M. M.; Sebastiani, F.; Alia, R. & Vendramin, G. G. (2007), 'Range-wide phylogeography and gene zones in Pinus pinaster Ait. revealed by chloroplast microsatellite markers', Molecular Ecology 16, 2137-2153.

Bues, R.; Eizaguirre, M.; Toubon, J. F. & Albages, R. (1996), 'Population generic structure and ecological differences among nine populations of Sesamia nonagrioides Lefebre (Lepidoptera: Noctuidae) from the western basin.', Canadian Entomologist 128(5), 849-858.

Busack, S. D.; Lawson, R. & Arjo, W. M. (2005), 'Mitochondrial DNA, allozymes, morphology and historical biogeography in the Podarcis vaucheri (Lacertidae) species complex', Amphibia-Reptilia 26, 239-256.

Canestrelli, D.; Cimmaruta, R.; Costantini, V. & Nascetti, G. (2006), 'Genetic diversity and phylogeography of the Apennine yellow-bellied toad Bombina pachypus, with implications for conservation', Molecular Ecology 15(12), 3741-3754.

Canestrelli, D.; Cimmaruta, R. & Nascetti, G. (2008), 'Population genetic structure and diversity of the Apennine endemic stream frog, Rana italica – insights on the Pleistocene evolutionary history of the Italian peninsular biota', Molecular Ecology 17, 3856-3872.

Canestrelli, D.; Cimmaruta, R. & Nascetti, G. (2007), 'Phylogeography and historical demography of the Italian treefrog, Hyla intermedia, reveals multiple refugia, population expansions and secondary contacts within peninsular Italy', Molecular Ecology 16(22), 4808-4821.

Capula, M. (1996), 'Evolutionary genetics of the insular lacertid lizard Podarcis tiliguerta: Genetic structure and population heterogeneity in a geographically fragmented species', Heredity 77, 518-529.

Caujapé-Castells, J. & Jansen, R. K. (2003), 'The influence of the Miocene Mediterranean desiccation on the geographical expansion and genetic variation of Androcymbium gramineum (Cav.) McBride (Colchicaceae)', Molecular Ecology 12, 1515-1525.

Cengel, B.; Velioglu, E.; Tolun, A. A. & Kaya, Z. (2000), 'Pattern and magnitude of genetic diversity in Pinus nigra ARNOLD subspecies pallasiana populatoins from Kazdagi: implications for in situ conservation', Silvae Genetica 49, 249-256.

Cheddadi, R.; Fady, B.; François, L.; Hajar, L.; Suc, J.-P.; Huang, K.; Demarteau, M.; Vendramin, G. G. & Ortu, E. (2009), 'Putative glacial refugia of Cedrus atlantica deduced from Quaternary pollen records and modern genetic diversity', Journal of Biogeography 36(7), 1361-1371.

Cleary, D. F. R.; Descimon, H. & Menken, S. B. J. (2002), 'Genetic and ecological differentiation between the butterfly sisterspecies Colias alfacariensis and Colias hyale', Contributions To Zoology 71.

Coart, E.; Van Glabeke, S.; Petit, R. J.; Van Bockstaele, E. & Roldan-Ruiz, I. (2005), 'Range wide versus local patterns of genetic diversity in hornbeam (Carpinus betulus L.)', Conservation Genetics 6(2), 259-273.

Comes, H. P. & Abbott, R. J. (1999), 'Population genetic structure and gene flow across arid versus mesic environments: A comparative study of two parapatric Senecio species from the Near East', Evolution 53(1), 36-54.

Comes, H. P. & Abbott, R. J. (1998), 'The relative importance of historical events and gene flow on the population structure of a Mediterranean ragwort, Senecio gallicus (Asteraceae)', Evolution 52(2), 355-367.

Conkle, M. T.; Schiller, G. & Grunwald, C. (1988) Electrophoretic analysis of diversity and phylogeny of Pinus brutia and closely related taxa Systematic Botany, 13, 411-424 

Cozzolino, S.; Cafasso, D.; Pellegrino, G.; Musacchio, A. & Widmer, A. (2003), 'Fine-scale phylogeographical analysis of Mediterranean Anacamptis palustris (Orchidaceae) populations based on chloroplast minisatellite and microsatellite variation', Molecular Ecology 12(10), 2783-2792.

Cronberg, N. (2000), 'Genetic diversity of the epiphytic bryophyte Leucodon sciuroides in formerly glaciated versus nonglaciated parts of Europe', Heredity 84, 710-720.

Cubas, P.; Pardo, C. & Tahiri, H. (2005), 'Genetic variation and relationships among Ulex (fabaceae) species in southern spain and northern morocco assessed by chloroplast microsatellite (cpSSR) markers1', American Journal of Botany 92, 2031-2043.

Deffontaine, V.; Libois, R.; Kotlik, P.; Sommer, R.; Nieberding, C.; Paradis, E.; Searle, J. B. & Michaux, J. (2005), 'Beyond the Mediterranean peninsulas: evidence of central European glacial refugia for a temperate forest mammal species, the bank vole (Clethrionomys glareolus)', Molecular Ecology 14(6), 1727-1739.

Ducci, F.; Proietti, R. & Favre, J. M. (1999), 'Allozyme assessment of genetic diversity within the relic Sicilian fir Abies nebrodensis (Lojac.) Mattei', Annals of Forest Science 56(4), 345-355.

ElenaRossello, J. A. & Cabrera, E. (1996), 'Isozyme variation in natural populations of cork-oak (Quercus suber L) - Population structure, diversity, differentiation and gene flow', Silvae Genetica 45(4), 229-235.

Escudero, M.; Vargas, P.; Arens, P.; Ouborg, N. J. & Luceño, M. (2009), 'The east-west-north colonization history of the Mediterranean and Europe by the coastal plant Carex extensa (Cyperaceae)', Molecular Ecology 19(9999), 352-370.

Fady, B.; Lefèvre, F.; Vendramin, G. G.; Ambert, A.; Régnier, C. & Bariteau, M. (2007), 'Genetic consequences of past climate and human impact on eastern Mediterranean Cedrus libani forests. Implications for their conservation', Conservation Genetics 9, 85-95.

Fallour, D.; Fady, B. & Lefevre, F. (1997) Study on isozyme variation in Pinus pinea L.: Evidence for low polymorphism Silvae Genetica, 46, 201-207 

Farley, R. A. & McNeilly, T. (2000), 'Diversity and divergence in Cistus salvifolius (L.) populations from contrasting habitats', Hereditas 132, 183-192.

Ferrazzini, D.; Monteleone, I. & Belletti, P. (2007), 'Genetic variability and divergence among Italian populations of common ash (Fraxinus excelsior L.)', Annals of Forest Science 64, 159-168.

Filippucci, M. G.; Rodino', E.; Nevo, E. & Capanna, E. (1988), 'Evolutionary genetics and systematics of the garden dormouse, Eliomys Wagner, 1840. 2-allozyme diversity and differentiation of chromosomal races', Bolletino di zoologia 55(1), 47-54.

Filippucci, M.-G.; Nascetti, G.; Capanna, E. & Bullini, L. (1987), 'Allozyme variation and systematics of European moles of the genus Talpa (Mammalia, Insectivora)', Journal of Mammalogy 68, 487-499.

Frati, F.; Hartl, G.; Lovari, S.; Delibes, M. & Markov, G. (1998), 'Quaternary radiation and genetic structure of the red fox Vulpes vulpes in the Mediterranean Basin, as revealed by allozymes and mitochondrial DNA', Journal of Zoology 245, 43-51.

Gantenbein, B. (2004), 'The genetic population structure of Buthus occitanus (Scorpiones : Buthidae) across the Strait of Gibraltar: calibrating a molecular clock using nuclear allozyme variation', Biological Journal of the Linnean Society 81(4), 519-534.

Gantenbein, B. & Largiader, C. R. (2002), 'Mesobuthus gibbosus (Scorpiones: Buthidae) on the island of Rhodes — hybridization between Ulysses' stowaways and native scorpions?', Molecular Ecology 11, 925-938.

Gantenbein, B.; Soleglad, M. E. & Fet, V. (2001), 'Euscorpius balearicus Caporiacco, 1950, stat. nov (Scorpiones : Euscorpiidae): molecular (allozymes and mtDNA) and morphological evidence for an endemic Balearic Islands species', Organisms Diversity & Evolution 1(4), 301-320.

Garnier, S.; P, A.; Audiot, P.; Prieur, B. & Rasplus, J.-Y. (2004), 'Isolation by distance and sharp discontinuities in gene frequencies: implications for the phylogeography of an alpine insect species, Carabus solieri', Molecular Ecology 13, 1883-1897.

Godoy, J. A.; Negro, J. J.; Hiraldo, F. & Donazar, J. A. (2004), 'Phylogeography, genetic structure and diversity in the endangered bearded vulture (Gypaetus barbatus, L.) as revealed by mitochondrial DNA', Molecular Ecology 13(2), 371-390.

Gomez, A.; González-Martínez, S. C.; Collada, C.; Climent, J. & Gil, L. (2003), 'Complex population genetic structure in the endemic Canary Island pine revealed using chloroplast microsatellite markers', Theoretical and Applied Genetics 107, 1123-1131.

Gomez, A.; Vendramin, G. G.; González-Martínez, S. C. & Alía, R. (2005), 'Genetic diversity and differentiation of two Mediterranean pines (Pinus halepensis Mill. and Pinus pinaster Ait.) along a latitudinal cline using chloroplast microsatellite markers', Diversity and Distributions 11, 257-263.

Gomory, D.; Paule, L. & Vysny, J. (2007), 'Patterns of allozyme variation in western Eurasian Fagus', Botanical Journal of the Linnean Society 154, 165-174.

Grant, O. M.; McNeilly, T. & Incoll, L. D. (2006), 'Genetic diversity of Cistus albidus in south-east Spain does not relate to mesoclimate', Functional Plant Biology 33(3), 247-255.

Grill, A.; Raijmann, L. E. L.; Van Ginkel, W.; Gkioka, E. & Menken, S. B. J. (2007), 'Genetic differentiation and natural hybridization between the Sardinian endemic Maniola nurag and the European Maniola jurtina', Journal of Evolutionary Biology 20, 1255-1270.

Griswold, C. K. & Baker, A. J. (2002), 'Time to the most recent common ancestor and divergence times of populations of common Chaffinches (Fringilla coelebs) in Europe and north Africa: insights into pleistocene refugia and current levels of migration', Evolution 56, 143-153.

Grundmann, M.; Ansell, S. W.; Russell, S. J.; Koch, A. M. & Vogel, J. C. (2007), 'Genetic structure of the widespread and common Mediterranean bryophyte Pleurochaete squarrosa (Brid.) Lindb. (Pottiaceae) - evidence from nuclear and plastidic DNA sequence variation and allozymes', Molecular Ecology 16, 709-722.

Gulbaba, A.G., Velioglu, E., Ozer, A.S., Dogan, B., Doerksen, A.H. and Adams, W.T. (1998) Population genetic structure of Kazdag¢ Fir (Abies equitorjani Ashers. et Sint), a narrow endemic to Turkey. Implication for in situ conservation. In: Zencirci, N., Kaya, Z., Anikster, Y. and Adams, W.T. (eds) Proceedings of International Symposium on In situ Conservation of Plant Genetic Diversity. CRIFC, Ankara, Turkey, pp. 271–281.

Haddioui, A. & Baaziz, M. (2001), 'Genetic diversity of natural populations of Atriplex halimus L. in Morocco: An isoenzyme-based overview', Euphytica 121, 99-106.

Horn, A.; Roux-Morabito, G.; Lieutier, F. & Kerdelhue, C. (2006), 'Phylogeographic structure and past history of the circum-Mediterranean species Tomicus destruens Woll. (Coleoptera : Scolytinae)', Molecular Ecology 15(6), 1603-1615.

Hunt, H. V.; Ansell, S. W.; Russell, S. J.; Schneider, H. & Vogel, J. C. (2009), 'Genetic diversity and phylogeography in two diploid ferns, Asplenium fontanum subsp. fontanum and A. petrarchae subsp. bivalens, in the western Mediterranean', Molecular Ecology 18(23), 4940-4954.

Jimenez, P.; Agundez, D.; Alia, R. & Gil, L. (1999), 'Genetic variation in central and marginal populations of Quercus suber L', Silvae Genetica 48(6), 278-284.

Kerdelhue, C.; Roux-Morabito, G.; Forichon, J.; Chambon, J.-M.; Robert, A. & Lieutier, F. (2002), 'Population genetic structure of Tomicus piniperda L. (Curculionidae: Scolytinae) on different pine species and validation of T. destruens (Woll.)', Molecular Ecology 11, 483-494.

Klein, M. & Seitz, A. (1994), 'Geographic differentiation between populations of Rhinocyllus conicus Frölich (Coleoptera: Curculionidae): concordance of allozyme and morphometric analysis', Zoological Journal of the Linnean Society 110(2), 181-191.

Klossa-Kilia, E.; Kilias, G. & Sfenthourakis, S. (2005), 'Increased genetic diversity in Greek populations of the genus Ligidium (Crustacea: Isopoda: Oniscidea) revealed by RFLP analysis of mtDNA segments', Contributions To Zoology 74, 255-264.

Korol, L.; Kara, N.; Isik, K. & Schiller, G. (1997), 'Genetic differentiation among and within natural and planted Cupressus sempervirens L. eastern Mediterranean populations', Silvae Genetica 46(2-3), 151-155.

Korol, L.; Shklar, G. & Schiller, G. (2002) Diversity among circum-Mediterranean populations of Aleppo pine and their differentiation from Brutia pine in their isoenzymes: additional results. Silvae Genetica, 51, 35-41 

Korol, L.; Shklar, G. & Schiller, G. (2002) Genetic variation within and among Pinus brutia Ten. seed stands in Turkey in their isozymes. Forest Genetics, 9, 233-242 

Lazaro, A. & Aguinagalde, I. (1998), 'Genetic diversity in Brassica oleracea L. (Cruciferae) and wild relatives (2n = 18) using isozymes', Annals of Botany 82, 821-828.

Lorenzini, R. & Lovari, S. (2006), 'Genetic diversity and phylogeography of the European roe deer: the refuge area theory revisited', Biological Journal of the Linnean Society 88(1), 85-100.

Lozier, J. D.; Roderick, G. K. & Mills, N. J. (2008), 'Evolutionarily significant units in natural enemies: Identifying regional populations of Aphidius transcaspicus (Hymenoptera: Braconidae) for use in biological control of mealy plum aphid', Biological Control 46(3), 532-541.

Lumaret, R.; Mir, C.; Michaud, H. & Raynal, V. (2002), 'Phylogeographical variation of chloroplast DNA in holm oak (Quercus ilex L.)', Molecular Ecology 11(11), 2327-2336.

Lumaret, R.; Ouazzani, N.; Michaud, H.; Vivier, G.; Deguilloux, M. F. & Di Giusto, F. (2004), 'Allozyme variation of oleaster populations (wild olive tree) (Olea europaea L.) in the Mediterranean Basin', Heredity 92(4), 343-351.

Lunt, D. H.; Ibrahim, K. M. & Hewitt, G. M. (1998), 'mtDNA phylogeography and postglacial patterns of subdivision in the meadow grasshopper Chorthippus parallelus', Heredity 80, 633-641.

Maliouchenko, O.; Palmé, A. E.; Buonamici, A.; Vendramin, G. G. & Lascoux, M. (2007), 'Comparative phylogeography and population structure of European Betula species, with particular focus on B. pendula and B. pubescens', Journal of Biogeography 34, 1601-1610.

Mantovani, B. & Scali, V. (1993), 'Genetic structure and phyletic relationships of eastern mediterranean Bacillus atticus Brunner (Insecta Phasmatodea): A biochemical study', Biochemical Genetics 31, 343-362.

Mantovani, B. & Scali, V. (1991), 'Allozymic characterization of Sardinian Bacillus rossius (Rossi) and B. atticus Brunner (Insecta Phasmatodea)', Genetica 83(3), 275-287.

Margaritopoulos, J. T.; Gotosopoulos, B.; Mamuris, Z.; Skouras, P. J.; Voudouris, K. C.; Bacandritsos, N.; Fantinou, A. A. & Tsitsipis, J. A. (2007), 'Genetic variation among Mediterranean populations of Sesamia nonagrioides (Lepidoptera: Noctuidae) as revealed by RFLP mtDNA analysis', Bulletin of Entomological Research 97, 299-308.

Marzouki, H.; Nasri, N.; Jouaud, B.; Bonnet, C.; Khaldi, A.; Bouzid, S. & Fady, B. (2009), 'Population Genetic Structure of Laurus nobilis L. Inferred From Transferred Nuclear Microsatellites', Silvae Genetica 58, 270-276.

Mengoni, A.; Selvi, F.; Cusimano, N.; Galardi, F. & Gonnelli, C. (2006), 'Genetic diversity inferred from AFLP fingerprinting in populations of Onosma echioides (Boraginaceae) from serpentine and calcareous soils', Plant Biosystems 140, 211-219.

Nascetti, G.; Cimmaruta, R.; Lanza, B. & Bullini, L. (1996), 'Molecular Taxonomy of European Plethodontid Salamanders (Genus Hydromantes)', Journal of Herpetology 30(2), 161-183.

Nevo, E.; Filippucci, M.-G. & Beiles, A. (1994), 'Genetic polymorphisms in subterranean mammals (Spalax ehrenbergi superspecies) in the Near East revisited: patterns and theory', Heredity 72, 465-487.

Nevo, E. & Yang, S. Y. (1979), 'Genetic diversity and climatic determinants of tree frogs in Israel', Oecologia 41(1), 47-63.

Nicholls, J. A.; Preuss, S.; Hayward, A.; Melika, G.; Csóka, G.; Nieves-aldrey, J.-l.; Askew, R. R.; Tavakoli, M.; Schönrogge, K. & Stone, G. N. (2010), 'Concordant phylogeography and cryptic speciation in two Western Palaearctic oak gall parasitoid species complexes', Molecular Ecology 19(9999), 592-609.

Nikolic, D. & Tucic, N. (1983) Isozyme variation within and among populations of European black pine (Pinus nigra Arnold) Silvae Genetica, 32, 80-89 

Orellana, M.; Blanché, C.; Simon, J. & Bosch, M. (2009), 'Genetic Diversity Within and among Disjunct Populations of the Mediterranean Island Endemic Delphinium pictum and D. requienii (Ranunculaceae)', Folia Geobotanica 44(1), 47-63.

Orellana, M.; Lopez-Pujol, J.; Blanché, C.; Rovira, A. & Bosch, M. (2008), 'Genetic diversity in Delphinium staphisagria (Ranunculaceae), a rare Mediterranean dysploid larkspur with medicinal uses', Genetica 135, 221-232.

Orellana, M. R.; Lopez-Pujol, J.; Blanche, C. & Bosch, M. (2007), 'Genetic diversity in the endangered dysploid larkspur Delphinium bolosii and its close diploid relatives in the series Fissa of the Western Mediterranean area', Biological Journal of the Linnean Society 92(4), 773-784.

Ortiz, M. A.; Tremetsberger, K.; Talavera, S.; Stuessy, T. & Garcia-Castano, J.-L. (2007), 'Population structure of Hypochaeris salzmanniana DC. (Asteraceae), an endemic species to the Atlantic coast on both sides of the Strait of Gibraltar, in relation to Quaternary sea level changes', Molecular Ecology 16, 541-552.

Ortiz-Dorda, J.; Martinez-Mora, C.; Correal, E.; Simon, B. & Cenis, J. L. (2005), 'Genetic Structure of Atriplex halimus Populations in the Mediterranean Basin', Annals of Botany 95, 827-834.

Oukil, S.; Bues, R.; Toubon, J.-F. & Quilici, S. (2002), 'Allozyme polymorphism in populations of Ceratitis capitata from Algeria, the northwestern Mediterranean coast and Reunion Island', Fruits 57, 183-191.

Palmé, A. E.; Semerokov, V. & Lascoux, M. (2003), 'Absence of geographical structure of chloroplast DNA variation in sallow, Salix caprea L.', Heredity 91, 465-474.

Palmé, A. E.; Su, Q.; Rautenberg, A.; Manni, F. & Lascoux, M. (2003), 'Postglacial recolonization and cpDNA variation of silver birch, Betula pendula', Molecular Ecology 12, 201-212.

Panetsos, K. P.; Aravanopoulos, F. A. & Scaltsoyiannes, A. (1998) Genetic variation of Pinus brutia from islands of the northeastern Aegean Sea. Silvae Genetica, 47, 115-120 

Papageorgiou, A. C.; Panetsos, K. P. & Hattemer, H. H. (1994) Genetic differentiation of natural Mediterranean cypress (Cupressus sempervirens L.) populations in Greece Forest Genetics, 1, 1-12 

Parducci, L.; Szmidt, A. E.; Madaghiele, A.; Anzidei, M. & Vendramin, G. G. (2001), 'Genetic variation at chloroplast microsatellites (cpSSRs) in Abies nebrodensis (Lojac.) Mattei and three neighboring Abies species', Theoretical And Applied Genetics 102(5), 733-740.

Pellegrino, G.; Palermo, A. M.; Noce, M. E.; Belliusci, F. & Musacchio, A. (2007), 'Genetic population structure in the Mediterranean Serapias vomeracea, a nonrewarding orchid group. Interplay of pollination strategy and stochastic forces?', Plant Systematics and Evolution 263, 145-157.

Petit, R. J.; Csaikl, U.; Bordacs, S.; Burg, K.; Coart, E.; Cottrell, J.; van Dam, B.; Deans, J.; Dumolin-Lapegue, S.; Fineschi, S.; Finkeldey, R.; Gillies, A.; Glaz, I.; Goicoechea, P. G.; Jensen, J. S.; König, A. O.; Lowe, A. J.; Madsen, S. F.; Matyas, G.; Munro, R. C.; Tabbener, H.; Taurchini, D.; de Vries, S. G. M.; Ziegenhagen, B. & Kremer, A. (2002), 'Chloroplast DNA variation in European white oaks Phylogeography and patterns of diversity based on data from over 2600 populations', Forest Ecology and Management 156, 5-26.

Pigliucci, M.; Benedettelli, S. & Villani, F. (1990), 'Spatial patterns of genetic-variability in italian chestnut (Castanea sativa)', Canadian Journal of Botany-Revue Canadienne De Botanique 68(9), 1962-1967.

Pineiro, R.; Fuertes Aguilar, J.; Drappermunt, D. & Nieto Feliner, G. (2007), 'Ecology matters: Atlantic-Mediterranean disjunction in the sand-dune shrub Armeria pungens (Plumbaginaceae)', Molecular Ecology 16, 2155-2171.

Pinho, C.; Harris, D. J. & Ferrand, N. (2003), 'Genetic Polymorphism of 11 Allozyme Loci in Populations of Wall Lizards (Podarcis sp.) From the Iberian Peninsula and North Africa', Biochemical Genetics 41, 343-359.

Prus-Glowacki, W. & Stephan, B. R. (1994) Genetic variation of Pinus sylvestris from Spain in relation to other European populations Silvae Genetica, 43, 7-14 

Prus-Glowacki, W.; Stephan, B. R.; Bujas, E.; Alia, R. & Marciniak, A. (2003), 'Genetic differentiation of autochthonous populations of Pinus sylvestris (Pinaceae) from the Iberian peninsula', Plant Systematics and Evolution 239, 55-66.

Queney, G.; Ferrand, N.; Weiss, S.; Mougel, F. & Monnerot, M. (2001), 'Stationary distributions of microsatellite loci between divergent population groups of the European rabbit (Oryctolagus cuniculus)', Molecular Biology and Evolution 18(12), 2169-2178.

Raddi, S. & Sumer, S. (1999), 'Genetic diversity in natural Cupressus sempervirens L. populations in Turkey', Biochemical Systematics and Ecology 27(8), 799-814.

Rasplus, J. Y.; Carcreff, E.; Cornuet, J. M. & Roques, A. (2000), Genetic structure of the cypress seed chalcid Megastigmus wachtli (Torymidae) within its Mediterranean distribution., in A.D. Austin & M. Dowton, ed., 'Hymenoptera: evolution, biodiversity and biological control', CSIRO Publishing, Collingwood, Australia, pp. 114-130.

Renau-Morata, B.; Nebauer, S. G.; Sales, E.; Allainguillaume, J.; Caligari, P. & Segura, J. (2005), 'Genetic diversity and structure of natural and managed populations of Cedrus atlantica (Pinaceae) assessed using random amplified polymorphic DNA', American Journal of Botany 92(5), 875-884.

Ribeiro, M. M.; Mariette, S.; Vendramin, G. G.; Szmidt, A. E.; Plomion, C. & Kremer, A. (2002), 'Comparison of genetic diversity estimates within and among populations of maritime pine using chloroplast simple-sequence repeat and amplified fragment length polymorphism data', Molecular Ecology 11, 869-877.

Ribeiro, M. M.; Plomion, C.; Petit, R.; Vendramin, G. G. & Szmidt, A. E. (2001), 'Variation in chloroplast single-sequence repeats in Portuguese maritime pine (Pinus pinaster Ait.)', Theoretical and Applied Genetics 102, 97-103.

Robledo-Arnuncio, J. J.; Collada, C.; Alía, R. & Gil, L. (2005), 'Genetic structure of montane isolates of Pinus sylvestris L. in a Mediterranean refugial area', Journal of Biogeography 32, 595-605.

Rokas, A.; Atkinson, R. J.; Webster, L.; Csoka, G. & Stone, G. N. (2003), 'Out of Anatolia: longitudinal gradients in genetic diversity support an eastern origin for a circum-Mediterranean oak gallwasp Andricus quercustozae', Molecular Ecology 12(8), 2153-2174.

Rossiter, S. J.; Benda, P.; Dietz, C.; Zhang, S. & Jones, G. (2007), 'Rangewide phylogeography in the greater horseshoe bat inferred from microsatellites: implications for population history, taxonomy and conservation', Molecular Ecology 16, 4699-4714.

Ruedi, M.; Walter, S.; Fischer, M. C.; Scaravelli, D.; Excoffier, L. & Heckel, G. (2008), 'Italy as a major Ice Age refuge area for the bat Myotis myotis (Chiroptera: Vespertilionidae) in Europe', Molecular Ecology 17, 1801-1814.

Sagnard, F.; Barberot, C. & Fady, B. (2002) Structure of genetic diversity in Abies alba Mill. from southwestern Alps: multivariate analysis of adaptive and non-adaptive traits for conservation in France. Forest Ecology and Management, 157, 175-189

Salvador, L.; Alia, R.; Agundez, D. & Gil, L. (2000), 'Genetic variation and migration pathways of maritime pine (Pinus pinaster Ait) in the Iberian peninsula', Theoretical and Applied Genetics 100, 89-95.

Salvato, P.; Battisti, A.; Concato, S.; Masutti, L.; Patarnello, T. & Zane, L. (2002), 'Genetic differentiation in the winter pine processionary moth (Thaumetopoea pityocampa-wilkinsoni complex), inferred by AFLP and mitochondrial DNA markers', Molecular Ecology 11(11), 2435-2444.

Scaltsoyiannes, A. (1999), 'Allozyme differentiation and phylogeny of cedar species', Silvae Genetica 48(2), 61-68.

Scaltsoyiannes, a.; Panestos, K. P. & Zaragotas, D. (1990), Genetic variation of greek fir as determined by isozyme analysis and its relation to other Mediterranean firs, in 'Proceedings of the third E. E. C. meeting - Sapins méditerranéens adaptation, sélection et sylviculture', EEC, Avignon, pp. 99-117.

Schiller, G.; Conkle, M. T. & Grunwald, C. (1986) Local differentiation among Mediterranean populations of Aleppo pine in their isoenzymes Silvae Genetica, 35, 11-19 

Schiller, G.; Korol, L.; Ungar, E. D.; Zehavi, A.; Gil, S. L. & Climent, M. J. (1999) Canary island pine (Pinus canariensis Chr.Sm. ex Dc.). 1- Differentiation among native populations in their isoenzymes. Forest Genetics, 6, 257-276 

Seabra, S. G.; Quartau, J. A. & Bruford, M. W. (2009), 'Spatio-temporal genetic variation in sympatric and allopatric Mediterranean Cicada species (Hemiptera, Cicadidae)', Biological Journal of the Linnean Society 96(2), 249-265.

Seeman, M. T. & Dodd, R. S. (2008), 'Genetic variability and structure of the remnant natural populations of Cedrus libani (Pinaceae) of Lebanon', Tree Genetics & Genomes 4, 757-766.

Simonato, M.; Mendel, Z.; Kerdelhué, C.; Rousselet, J.; Magnoux, E.; Salvato, P.; Roques, A.; Battisti, A. & Zanz, L. (2007), 'Phylogeography of the pine processionary moth Thaumetopoea wilkinsoni in the Near East', Molecular Ecology 16, 2273-2283.

Soranzo, N.; Alia, R.; Provan, J. & Powell, W. (2000), 'Patterns of variation at a mitochondrial sequence-tagged site locus provides new insights into the postglacial history of European Pinus sylvestris populations', Molecular Ecology 9, 1205-1211.

Spagnuolo, V.; Muscariello, L.; Cozzolino, S.; Castaldo Cobianchi, R. & Giordano, S. (2007), 'Ubiquitous genetic diversity in ISSR markers between and within populations of the asexually producing moss Pleurochaete squarrosa', Plant Ecology 188, 91-101.

Spagnuolo, V.; Terracciano, S. & Giordano, S. (2009), 'Clonal diversity and geographic structure in Pleurochaete squarrosa (Pottiaceae): different sampling scale approach', Journal of Plant Research 122(2), 161-170.

Stamatis, C.; Suchentrunk, F.; Moutou, K. A.; Giacometti, M.; Haerer, G.; Djan, M.; Vapa, L.; Vukovic, M.; Tvrtkovi0107, N.; Sert, H.; Alves, P. C. & Mamuris, Z. (2009), 'Phylogeography of the brown hare (Lepus europaeus) in Europe: a legacy of south-eastern Mediterranean refugia?', Journal of Biogeography 36(9999), 515-528.

Stone, G. N.; Atkinson, R. J.; Rokas, A.; Csoka, G. & Nieves-Aldrey, J.-L. (2001), 'Differential success in northwards range expansion between ecotypes of the marble gallwasp Andricus kollari: a tale of two lifecycles', Molecular Ecology 10, 761-778.

Suchentrunk, F.; Mamuris, Z.; Sfougaris, A. I. & Stamatis, C. (2003), 'Biochemical Genetic Variability in Brown Hares (Lepus europaeus) from Greece', Biochemical Genetics 41(5), 127-140.

Teisseire, H.; Fady, B. & Pichot, C. (1995), 'Allozyme variation in five French populations of Aleppo pine (Pinus halepensis Miller)', Forest Genetics 2(4), 227-236.

Terrab, A.; Paun, O.; Talavera, S.; Tremetsberger, K.; Arista, M. & Stuessy, T. F. (2006), 'Genetic diversity and population structure in natural populations of Moroccan atlas cedar (Cedrus atlantica; Pinaceae) determined with cpSSR markers', American Journal of Botany 93(9), 1274-1280.

Terrab, A.; Schönswetter, P.; Talavera, S.; Vela, E. & Stuessy, T. F. (2008), 'Rangewide phylogeography of Juniperus thurifera L., a presumptive keystone species of Western Mediterraean vegetation during cold stages of the Pleistocene', Molecular Phylogenetics and Evolution EOP, EOP.

Tolun, A. A.; Velioglu, E.; CEngel, B. & Kaya, Z. (2000), 'Genetic Structure of Black Pine (Pinus nigra Arnold subspecies pallasiana) Populations Sampled from the Bolkar Mountains', Silvae Genetica 49, 113-119.

Tyler, T. (2004), 'Studies in the Melica ciliata-complex: 1. Distribution of allozyme variation within and among individuals, populations and geographic regions', Plant Systematics and Evolution 248(1-4), 1-30.

Vasconcelos, T.; Horn, A.; Lieutier, F.; Branco, M. & Kerdelhue, C. (2006), 'Distribution and population genetic structure of the Mediterranean pine shoot beetle Tomicus destruens in the Iberian Peninsula and Southern France', Agricultural and Forest Entomology 8(2), 103-111.

Velo-Anton, G.; Garcia-Paris, M. & Cordero Rivera, A. (2008), 'Patterns of nuclear and mitochondrial DNA variation in Iberian populations of Emys orbicularis (Emydidae): conservation implications', Conservation Genetics 9(5), 1263-1274.

Vendramin, G. G.; Anzidel, M.; Madaghiele, A. & Bucci, G. (1998), 'Distribution of genetic diversity in Pinus pinaster Ait. as revealed by chloroplast microsatellites', Theoretical and Applied Genetics 97, 456-463.

Vendramin, G. G.; Degen, B.; Petit, R. J.; Anzidei, M.; Madaghiele, A. & Ziegenhagen, B. (1999), 'High level of variation at Abies alba chloroplast microsatellite loci in Europe', Molecular Ecology 8, 11171126.

Vendramin, G. G.; Fady, B.; Gonzalez-Martinez, S. C.; Hu, F. S.; Scotti, I.; Sebastiani, F.; Soto, A.; Petit, R. J. & Kohn, J. (2008), 'Genetically Depauperate But Widespread: The Case Of An Emblematic Mediterranean Pine', Evolution 62(3), 680-688.

Vicario, F.; Vendramin, G.; Rossi, P.; Lio, P. & Giannini, R. (1995) Allozyme, chloroplast DNA and RAPD markers for determining genetic relationships between Abies alba and the relic population of Abies nebrodensis Theoretical and Applied Genetics, 90, 1012-1018 

Vila, M.; Vidal-Romani, J. R. & Bjorklund, M. (2005), 'The importance of time scale and multiple refugia: Incipient speciation and admixture of lineages in the butterfly Erebia triaria (Nymphalidae)', Molecular Phylogenetics and Evolution 36, 249-260.

Villani; Sansotta; Cherubini; Cesaroni & Sbordoni (1999), 'Genetic structure of natural populations of Castanea sativa in Turkey: evidence of a hybrid zone', Journal of Evolutionary Biology 12(2), 233-244.

Vitalis, R.; Riba, M.; Colas, B.; Grillas, P. & Olivieri, I. (2002), 'Multilocus genetic structure at contrasted spatial scales of the endangered water fern Marsilea strigosa Willd. (Marsileaceae, Pteridophyta)', American Journal of Botany 89, 1142-1155.

Wahid, N.; Gonzalez-Martinez, S. C.; El Hadrami, I. & Boulli, A. (2004), 'Genetic structure and variability of natural populations of maritime pine (Pinus pinaster Aiton) in Morocco', Silvae Genetica 53(3), 93-99.

Westberg, E. & Kadereit, J. W. (2008), 'The influence of sea currents, past disruption of gene flow and species biology on the phylogeographical structure of coastal flowering plants', Journal of Biogeography 36, 1398-1410.

Yacine, A. & Lumaret, R. (1989), 'Genetic Diversity In Holm-Oak (Quercus ilex L) - Insight From Several Enzyme Markers', Silvae Genetica 38(3-4), 140-148.

Zangari, F.; Cimmaruta, R. & Nascetti, G. (2006), 'Genetic relationships of the western Mediterranean painted frogs based on allozymes and mitochondrial markers: evolutionary and taxonomic inferences (Amphibia, Anura, Discoglossidae)', Biological Journal of the Linnean Society 87, 515-538.
