# Supplementary material for: Large-scale longitudinal gradients of genetic diversity: a meta-analysis across six phyla in the Mediterranean basin
Source: Ecol Evol. 2012 Sep 14;2(10):2600–14. doi: 10.1002/ece3.350 (PMC3492785; doi:10.1002/ece3.350)
Supplement: Supplementary file 3 [file ece30002-2600-SD3.rtf]

Supplementary material II
Testing for biases related to non-independence of data.

Seventy percent of the studies in our primary dataset reported several metrics (e.g. allelic richness, percentage of polymorphic loci and heterozygosity) to describe the genetic diversity of populations for a given species. Therefore, the dataset of summary-effects might suffer from redundancy. Non-independence of the effect-sizes may affect the Type I error rate as well as the precision of estimations (Hartung et al. 2008). Treatment of such non-independent multivariate data can be done (1) by performing separate analyses, (2) by weighting each outcome by the inverse of its frequency or (3) by summarizing the outcomes using data reduction procedures (e.g. a principal component analysis). Prior to such treatments, the effect of redundancy can be explored through sensitivity analysis using randomization procedures (Hartung et al., 2008).

As a first step, we evaluated the redundancy of outcomes using two resampling procedures after which we calculated Zr. We resampled the dataset by randomly choosing (1000 times) a single effect-size per population at each iteration cycle, thus eliminating redundancy. We performed a second resampling procedure by randomly picking the same number of GDpop measures per population in each study as in the original dataset so that the 1000 resampled datasets had the same number of effect-sizes as the initial dataset. Whatever the resampling strategy used, all Zr remained significantly positive and their confidence interval intercepted 0 in less than 1% of cases. 
As a second step and to be perfectly sure of the absence of effect of redundancy on effect sizes, we use a principal component analysis to reduce GDpop variables to one per population and per study. The summary effect size we then calculated was of the same magnitude and significance as the one generated without the PCA procedure (0.0508 [CI 0.0088 to 0.0929] with PCA vs 0.0688 [CI 0.039 to 0.098] without PCA).

As the redundancy affected neither the direction of the relationship between GDpop and longitude nor its significance, we decided to use the entire dataset in the following meta-analyses and to not perform any statistical treatment to reduce redundancy.
