# Supplementary material for: Large-scale longitudinal gradients of genetic diversity: a meta-analysis across six phyla in the Mediterranean basin
Source: Ecol Evol. 2012 Sep 14;2(10):2600–14. doi: 10.1002/ece3.350 (PMC3492785; doi:10.1002/ece3.350)
Supplement: Supplementary file 4 [file ece30002-2600-SD4.doc]

**Supplementary material IV**

**Effect size values used in figures 2 to 4**

Table IV-1. Effect sizes for partial correlation between genetic diversity and longitude (de-trended for latitude) of different datasets from the Mediterranean Basin. Fixed effect model. Metric used for the meta-analysis: Z-transformed Pearson's r. CI: lowest and highest value of the 95% confidence interval for mean effect. Q: Heterogeneity test value. p(Q): p-value of Q-test (Q is considered significant when p(Q) < 0.05)

| Effect tested | Dataset | | Mean Effect | CI low | CI high | Number of effect sizes tested | Q | p(Q) |
| --- | --- | --- | --- | --- | --- | --- | --- | --- |
| Biogeographical effect | Mediterranean Basin | | 0.069 | 0.039 | 0.098 | 428 | 1180.87 | 0.00 |
|  | Northern Mediterranean | | 0.058 | 0.027 | 0.090 | 399 | 1089.83 | 0.00 |
|  | Southern Mediterranean | | 0.171 | 0.052 | 0.286 | 53 | 98.59 | 0.00 |
|  | Mediterranean continent | | 0.102 | 0.069 | 0.135 | 377 | 1040.33 | 0.00 |
|  | Mediterranean islands | | 0.018 | -0.052 | 0.089 | 108 | 432.86 | 0.00 |
| Pylogenetic effect | kingdom | Animal | 0.140 | 0.09 | 0.202 | 159 | 495.03 | 0.00 |
|  |  | Plant | 0.042 | 0.007 | 0.077 | 269 | 677.06 | 0.00 |
|  | class | Bryophytes | -0.455 | -0.669 | -0.241 | 9 | 4.26 | 0.83 |
|  |  | Dicotyledones | 0.029 | -0.017 | 0.074 | 121 | 379.65 | 0.00 |
|  |  | Gymnosperms | 0.107 | 0.043 | 0.171 | 122 | 251.15 | 0.00 |
|  |  | Monocotyledones | 0.152 | -0.001 | 0.305 | 14 | 5.55 | 0.96 |
|  |  | Pteridophytes | -0.009 | -0.795 | 0.777 | 3 | 0.9 | 0.64 |
|  |  | Amphibians | 0.015 | -0.127 | 0.158 | 29 | 64.88 | 0.00 |
|  |  | Arachnida | 0.362 | 0.090 | 0.635 | 10 | 10.6 | 0.30 |
|  |  | Birds | 0.003 | -0.714 | 0.720 | 5 | 11.45 | 0.02 |
|  |  | Crustaceans | 0.561 | -5.793 | 6.914 | 2 | 0 | 0.96 |
|  |  | Gastropods | -0.963 | -1.791 | -0.135 | 3 | 0.06 | 0.97 |
|  |  | Insects | 0.239 | 0.159 | 0.318 | 69 | 308.99 | 0.00 |
|  |  | Mammals | 0.051 | -0.102 | 0.204 | 33 | 49.23 | 0.03 |
|  |  | Reptiles | 0.103 | -0.336 | 0.542 | 8 | 1.88 | 0.97 |
| Marker type effect | Nuclear | | 0.074 | 0.035 | 0.112 | 291 | 889.06 | 0.00 |
|  | Plastidial | | 0.060 | 0.003 | 0.120 | 64 | 182.91 | 0.00 |
|  | genomic | | 0.049 | -0.079 | 0.177 | 36 | 58.79 | 0.00 |
|  | mitochondrial | | 0.080 | -0.046 | 0.193 | 37 | 49.41 | 0.04 |

Table IV-2. Effect sizes for partial correlation between genetic diversity and longitude (de-trended for latitude) of different ecological, reproductive and life history traits in vascular plants. Fixed effect model. Metric used for the meta-analysis: Z-transformed Pearson's partial r. Geographical range according to Olson 2001. CI: lowest and highest value of the 95% confidence interval for mean effect. Q: Heterogeneity test value. p(Q): p-value of Q-test (Q is considered significant when p(Q) < 0.05)

| Effect tested | Dataset | Mean Effect | CI low | CI high | Number of effect sizes tested | Q | p(Q) |
| --- | --- | --- | --- | --- | --- | --- | --- |
| Ecological effect (temperature) | Thermo-Mediterranean (Th) | -0.01 | -0.14 | 0.11 | 27 | 75.75 | 0.00 |
|  | Meso-Mediterranean (Me) | 0.174 | 0.099 | 0.250 | 53 | 125.90 | 0.00 |
|  | Supra-Mediterranean (Su) | 0.060 | -0.012 | 0.126 | 39 | 78.63 | 0.00 |
|  | Mountain-Mediterranean (Mt) | -0.040 | -0.077 | 0.114 | 82 | 212.49 | 0.00 |
|  | Eurytherm | 0.003 | -0.025 | 0.070 | 64 | 388.32 | 0.00 |
| Seed dispersal effect | Wind-dispersed seeds (anemochory) | 0.143 | 0.088 | 0.198 | 147 | 376.25 | 0.00 |
|  | Animal-dispersed seeds (zoochory) | -0.02 | -0.07 | 0.03 | 90 | 232.88 | 0.00 |
|  | Gravity-dispersed seeds (barochory) | 0.164 | 0.041 | 0.329 | 17 | 7.46 | 0.92 |
|  | Water-dispersed seeds (hydrochory) | -0.009 | -0.795 | 0.777 | 3 | 0.90 | 0.64 |
|  | Non assigned dispersal mode | -0.434 | -0.629 | -0.240 | 12 | 6.02 | 0.87 |
| Pollen dissemination effect | Wind-dispersed pollen (anemogamy) | 0.103 | 0.062 | 0.144 | 174 | 431.20 | 0.00 |
|  | Insect-dispersed pollen (entomogamy) | -0.033 | -0.011 | 0.055 | 76 | 119.34 | 0.00 |
|  | Pollen dispersal type not assigned | -0.434 | -0.629 | -0.240 | 12 | 6.02 | 0.87 |
|  | Water-dispersed pollen (hydrogamy) | -0.260 | -0.488 | -0.030 | 7 | 6.36 | 0.38 |
| Life history trait effect (Raunkiaer types) | Geophyte | 0.115 | -0.050 | 0.280 | 13 | 4.99 | 0.96 |
|  | Phanerophyte | 0.074 | 0.030 | 0.120 | 192 | 487.57 | 0.00 |
|  | Hemicryptophyte | 0.029 | -0.100 | 0.240 | 23 | 31.55 | 0.09 |
|  | Chamephyte | -0.245 | -0.359 | -0.131 | 25 | 77.28 | 0.00 |
|  | Therophyte | 0.104 | -0.057 | 0.265 | 16 | 44.75 | 0.00 |

Table IV-3. Effect sizes for partial correlation between genetic diversity and longitude (de-trended for latitude) of different marker types and metrics of vascular plants. Fixed effect model. Metric used for the meta-analysis: Z-transformed Pearson's partial r. Geographical range according to Olson 2001. CI: lowest and highest value of the 95% confidence interval for mean effect. Q: Heterogeneity test value. p(Q): p-value of Q-test (Q is considered significant when p(Q) < 0.05)

| Effect tested | Dataset | Mean Effect | CI low | CI high | Number of effect sizes tested | Q | p(Q) |
| --- | --- | --- | --- | --- | --- | --- | --- |
| Marker type effect | Maternally inherited mitochondrial marker | 0.074 | -0.046 | 0.193 | 37 | 49.41 | 0.04 |
|  | Paternally inherited plastidial marker | 0.06 | 0.003 | 0.118 | 64 | 182.91 | 0.00 |
|  | Bi-parentally inherited nuclear marker | 0.07 | 0.044 | 0.113 | 291 | 889.06 | 0.00 |
|  | Genomic marker | 0.05 | -0.079 | 0.177 | 36 | 58.79 | 0.01 |
| Genetic diversity metric | Equitability (isozymes) | 0.01 | -0.061 | 0.072 | 96 | 255.62 | 0.00 |
|  | Richness (isozymes) | -0.03 | -0.120 | 0.074 | 49 | 120.87 | 0.00 |
