# Supplementary material for: Large-scale longitudinal gradients of genetic diversity: a meta-analysis across six phyla in the Mediterranean basin
Source: Ecol Evol. 2012 Sep 14;2(10):2600–14. doi: 10.1002/ece3.350 (PMC3492785; doi:10.1002/ece3.350)
Supplement: Supplementary file 5 [file ece30002-2600-SD5.rtf]

Supplementary material V
List of taxa found in primary studies with their reference code (see Supplementary material II) and taxonomical levels used as categorical moderators in the meta-analysis

Kingdom	Phylum	Class	Family	Genus_species	Bibliographic Reference Code	
animal	Arthropod	Arachnida	Buthidae	Androctonus australis	BenOthmen_2004	
animal	Arthropod	Arachnida	Buthidae	Buthus occitanus	BenOthmen_2004	
animal	Arthropod	Arachnida	Buthidae	Buthus occitanus	Gantenbein_2004	
animal	Arthropod	Arachnida	Buthidae	Buthus occitanus mardochei	Gantenbein_2004	
animal	Arthropod	Arachnida	Buthidae	Mesobuthus gibbosus	Gantenbein_2002	
animal	Arthropod	Arachnida	Euscorpiidae	Euscorpius balearicus	Gantenbein_2001	
animal	Arthropod	Arachnida	Euscorpiidae	Euscorpius flavicaudis	Gantenbein_2001	
animal	Arthropod	Crustacea	Crustacea	Ligidium spp.	KlossaKilia_2005	
animal	Arthropod	Insect	Acrididae	Chorthippus parellelus	Lunt_1998	
animal	Arthropod	Insect	Bacillaceae	Bacillus atticus	Mantovani_1993	
animal	Arthropod	Insect	Bacillaceae	Bacillus rossius	Mantovani_1991	
animal	Arthropod	Insect	Braconidae	Aphidius transcaspicus	Lozier_2008	
animal	Arthropod	Insect	Carabidae	Carabus solieri	Garnier_2004	
animal	Arthropod	Insect	Cicadidae	Cicada barbara	Seabra_2009	
animal	Arthropod	Insect	Cicadidae	Cicada orni	Seabra_2009	
animal	Arthropod	Insect	Curculionidae	Larinus cynarae	Briese_1996	
animal	Arthropod	Insect	Curculionidae	Rhinocyllus conicus	Klein_1994	
animal	Arthropod	Insect	Cynipidae	Andricus kollari	Stone_2001	
animal	Arthropod	Insect	Cynipidae	Andricus quercustozae	Rokas_2003	
animal	Arthropod	Insect	Dytiscidae	Nebrioporus baeticus	Abellan_2009	
animal	Arthropod	Insect	Dytiscidae	Nebrioporus ceresyi	Abellan_2009	
animal	Arthropod	Insect	Dytiscidae	Nebrioporus nemethi	Abellan_2009	
animal	Arthropod	Insect	Hydraenidae	Ochthebius glaber	Abellan_2009	
animal	Arthropod	Insect	Hydraenidae	Ochthebius notabilis	Abellan_2009	
animal	Arthropod	Insect	Noctuidae	Sesamia nonagrioides	Bues_1996, Margaritopoulos_2007	
animal	Arthropod	Insect	Notodontidae	Thaumetopoea pityocampa	Salvato_2002	
animal	Arthropod	Insect	Notodontidae	Thaumetopoea wilkinsoni	Simonato_2007	
animal	Arthropod	Insect	Nymphalidae	Erebia triara	Vila_2005	
animal	Arthropod	Insect	Nymphalidae	Maniola jurtina	Grill_2007	
animal	Arthropod	Insect	Nymphalidae	Maniola nurag	Grill_2007	
animal	Arthropod	Insect	Pieridae	Colias alfacariensis	Cleary_2002	
animal	Arthropod	Insect	Rhaphidophoridae	Dolichopoda schiavazzii schiavazzii	Allegrucci_1997	
animal	Arthropod	Insect	Scolytidae	Tomicus destruens	Horn_2006, Vasconcelos_2006	
animal	Arthropod	Insect	Scolytidae	Tomicus piniperda	Kerdelhue_2002	
animal	Arthropod	Insect	Tephritidae	Bactrocera oleae	Augustinos_2005	
animal	Arthropod	Insect	Tephritidae	Ceratitis capitata	Oukil_2002	
animal	Arthropod	Insect	Torymidae	Megastigmus dorsalis	Nicholls_2010	
animal	Arthropod	Insect	Torymidae	Megastigmus wachtli	Rasplus_2000	
animal	Chordata	Amphibian	Alytidae	Discoglossus jeanneae	Zangari_2006	
animal	Chordata	Amphibian	Alytidae	Discoglossus montalentii	Zangari_2006	
animal	Chordata	Amphibian	Alytidae	Discoglossus pictus auritus	Zangari_2006	
animal	Chordata	Amphibian	Alytidae	Discoglossus pictus pictus	Zangari_2006	
animal	Chordata	Amphibian	Alytidae	Discoglossus sardus	Zangari_2006	
animal	Chordata	Amphibian	Bombinatoridae	Bombina pachypus	Canestrelli_2006	
animal	Chordata	Amphibian	Hylidae	Hyla arborea	Nevo_1979	
animal	Chordata	Amphibian	Hylidae	Hyla intermedia	Canestrelli_2007	
animal	Chordata	Amphibian	Plethodontidae	Hydromantes ambrosii	Nascetti_1996	
animal	Chordata	Amphibian	Plethodontidae	Hydromantes genei	Nascetti_1996	
animal	Chordata	Amphibian	Plethodontidae	Hydromantes imperialis	Nascetti_1996	
animal	Chordata	Amphibian	Plethodontidae	Hydromantes italicus	Nascetti_1996	
animal	Chordata	Amphibian	Ranidae	Rana italica	Canestrelli_2008	
animal	Chordata	Amphibian	Salamandridae	Chioglossa lusitanica	Alexandrino_2000	
animal	Chordata	Bird	Accipitridae	Gypaetus barbatus	Godoy_2004	
animal	Chordata	Bird	Fringillidae	Fringilla coelebs	Griswold_2002	
animal	Chordata	Bird	Strigidae	Strix aluco	Brito_2005	
animal	Chordata	Mammal	Canidae	Vulpes vulpes	Frati_1998	
animal	Chordata	Mammal	Cervidae	Capreolus capreolus	Lorenzi_2006	
animal	Chordata	Mammal	Gliridae	Eliomys spp	Filippucci_1988	
animal	Chordata	Mammal	Leporidae	Lepus europaeus	Stamatis_2008, Suchentrunk_2003	
animal	Chordata	Mammal	Leporidae	Oryctolagus cuniculus	Queney_2001	
animal	Chordata	Mammal	Muridae	Apodemus sylvaticus	deBellocq_2005	
animal	Chordata	Mammal	Muridae	Spalax ehrenbergi	Nevo_1994	
animal	Chordata	Mammal	Rhinolophidae	Rhinolophus ferrumequinum	Rossiter_2007	
animal	Chordata	Mammal	Talpidae	Talpa sp	Filippuci_1987	
animal	Chordata	Mammal	Vespertilionidae	Myotis myotis	Ruedi_2008	
animal	Chordata	Reptile	Emydidae	Emys orbicularis	VeloAnton_2008	
animal	Chordata	Reptile	Lacertidae	Podarcis hispanica	Pinho_2003	
animal	Chordata	Reptile	Lacertidae	Podarcis tiliguerta	Capula_1996	
animal	Chordata	Reptile	Lacertidae	Podarcis vaucheri	Busack_2005	
animal	Molluscs	Gasteropod	Chondrinidae	Solatopupa similis	Boato_1988	
plant	Bryophyte	Bryophyte	Leucodontaceae	Leucodon sciuroides	Cronberg_2000	
plant	Bryophyte	Bryophyte	Pottiaceae	Pleurochaete squarrosa	Grundmann_2007, Spagnuolo_2007, Spagnuolo_2009	
plant	Pteridophyte	Pteridophyte	Marsileaceae	Marsilea strigosa	Vitalis_2002	
plant	Spermaphyte	Dicotyledone	Apiaceae	Eryngium maritimum	Westberg_2008	
plant	Spermaphyte	Dicotyledone	Aspleniaceae	Asplenium fontanum subsp. fontanum	Hunt_2009	
plant	Spermaphyte	Dicotyledone	Aspleniaceae	Asplenium petrarchae subsp. bivalens	Hunt_2009	
plant	Spermaphyte	Dicotyledone	Asteracea	Senecio vernalis	Comes_1999	
plant	Spermaphyte	Dicotyledone	Asteraceae	Hypochaeris salzmanniana	Ortiz_2007	
plant	Spermaphyte	Dicotyledone	Asteraceae	Senecio gallicus	Comes_1998	
plant	Spermaphyte	Dicotyledone	Asteraceae	Senecio glaucus	Comes_1999	
plant	Spermaphyte	Dicotyledone	Betulaceae	Betula pendula	Maliouchenko_2007, Palme_2003	
plant	Spermaphyte	Dicotyledone	Betulaceae	Carpinus orientalis	Coart_2005	
plant	Spermaphyte	Dicotyledone	Boraginaceae	Onosma echioides	Mengoni_2006	
plant	Spermaphyte	Dicotyledone	Brassicaceae	Brassica cretica	Lazaro_1998	
plant	Spermaphyte	Dicotyledone	Brassicaceae	Brassica montana	Lazaro_1998	
plant	Spermaphyte	Dicotyledone	Brassicaceae	Cakile maritima	Westberg_2008	
plant	Spermaphyte	Dicotyledone	Chenopodiaceae	Atriplex halimus	Haddioui_2001, OrtizDorda_2005	
plant	Spermaphyte	Dicotyledone	Cistaceae	Cistus albidus	Grant_2006	
plant	Spermaphyte	Dicotyledone	Cistaceae	Cistus salvifolius	Farley_2000	
plant	Spermaphyte	Dicotyledone	Cyperaceae	Carex extensa	Escudero_2009	
plant	Spermaphyte	Dicotyledone	Fabaceae	Ulex africanus	Cubas_2005	
plant	Spermaphyte	Dicotyledone	Fabaceae	Ulex baeticus	Cubas_2005	
plant	Spermaphyte	Dicotyledone	Fabaceae	Ulex borgiae	Cubas_2005	
plant	Spermaphyte	Dicotyledone	Fagaceae	Castanea sativa	Pigliucci_1990, Villani_1999	
plant	Spermaphyte	Dicotyledone	Fagaceae	Fagus sylvatica	Gomory_2007	
plant	Spermaphyte	Dicotyledone	Fagaceae	Quercus canariensis	Petit_2002 (PersComm)	
plant	Spermaphyte	Dicotyledone	Fagaceae	Quercus faginea	Petit_2002 (PersComm)	
plant	Spermaphyte	Dicotyledone	Fagaceae	Quercus frainetto	Petit_2002 (PersComm)	
plant	Spermaphyte	Dicotyledone	Fagaceae	Quercus ilex	Yacine_1989, Lumaret_2002	
plant	Spermaphyte	Dicotyledone	Fagaceae	Quercus mix	Petit_2002 (PersComm)	
plant	Spermaphyte	Dicotyledone	Fagaceae	Quercus petraea	Petit_2002 (PersComm)	
plant	Spermaphyte	Dicotyledone	Fagaceae	Quercus pubescens	Petit_2002 (PersComm)	
plant	Spermaphyte	Dicotyledone	Fagaceae	Quercus pyrenaica	Petit_2002 (PersComm)	
plant	Spermaphyte	Dicotyledone	Fagaceae	Quercus robur	Petit_2002 (PersComm)	
plant	Spermaphyte	Dicotyledone	Fagaceae	Quercus suber	ElenaRossello_1996, Jimenez_1999, Petit_Guichoux (PersComm)	
plant	Spermaphyte	Dicotyledone	Lamiaceae	Phlomis crinita subsp. Crinita	Albaladejo_2007	
plant	Spermaphyte	Dicotyledone	Lamiaceae	Phlomis crinita subsp. Malacitana	Albaladejo_2007	
plant	Spermaphyte	Dicotyledone	Lamiaceae	Phlomis lychnitis	Albaladejo_2007	
plant	Spermaphyte	Dicotyledone	Lauraceae	Laurus nobilis	Marzouki_2009	
plant	Spermaphyte	Dicotyledone	Myrtaceae	Myrtus communis	Agrimonti_2007	
plant	Spermaphyte	Dicotyledone	Myrtaceae	Myrtus communis	Bruna_2007	
plant	Spermaphyte	Dicotyledone	Oleaceae	Fraxinus angustifolia	Vendramin (PersComm)	
plant	Spermaphyte	Dicotyledone	Oleaceae	Fraxinus excelsior	Ferrazzini_2007	
plant	Spermaphyte	Dicotyledone	Oleaceae	Fraxinus ornus	Vendramin (PersComm)	
plant	Spermaphyte	Dicotyledone	Oleaceae	Olea europaea	Lumaret_2004, Breton_2006, Belaj_2007	
plant	Spermaphyte	Dicotyledone	Plumbaginaceae	Armeria pungens	Pineiro_2007	
plant	Spermaphyte	Dicotyledone	Primulaceae	Cyclamen balearicum	Affre_1997	
plant	Spermaphyte	Dicotyledone	Ranunculaceae	Delphinium fissum ssp. Sordidum	Orellana_2007	
plant	Spermaphyte	Dicotyledone	Ranunculaceae	Delphinium pictum	Orellana_2009	
plant	Spermaphyte	Dicotyledone	Ranunculaceae	Delphinium staphisagria	Orellana_2008	
plant	Spermaphyte	Dicotyledone	Salicaceae	Salix caprea	Palme_2003b	
plant	Spermaphyte	Gymnosperm	Cupressaceae	Cupressus atlantica	Bechir_2004	
plant	Spermaphyte	Gymnosperm	Cupressaceae	Cupressus sempervirens	Papageorgiou_1994, Korol_1997, Raddi_1999, Bagnoli_2009, Fady (PersComm)	
plant	Spermaphyte	Gymnosperm	Cupressaceae	Juniperus phoenicea	Boratynski_2009	
plant	Spermaphyte	Gymnosperm	Cupressaceae	Juniperus thurifera	Terrab_2008	
plant	Spermaphyte	Gymnosperm	Pinaceae	Abies alba	Vicario_1995, Ducci_1999, Fady_1999, Vendramin_1999, Sagnard_2002	
plant	Spermaphyte	Gymnosperm	Pinaceae	Abies cephalonica	Scaltsoyiannes_1990, Fady_1993, Parducci_2001a	
plant	Spermaphyte	Gymnosperm	Pinaceae	Abies equi-trojani	Gulbaba_1996	
plant	Spermaphyte	Gymnosperm	Pinaceae	Cedrus atlantica	Scaltsoyiannes_1999b, RenauMoarat_2005, Terrab_2006, Cheddadi_2009	
plant	Spermaphyte	Gymnosperm	Pinaceae	Cedrus libani	Scaltsoyiannes_1999b, BouDagherKharrat_2007, Fady_2007, Fady_2008, Seeman_2008	
plant	Spermaphyte	Gymnosperm	Pinaceae	Pinus brutia	Schiller1986, Conkle1988, Bucci_1998, Panetsos_1998, Korol_2002b	
plant	Spermaphyte	Gymnosperm	Pinaceae	Pinus canariensis	Schiller_1999, Gomez_2003	
plant	Spermaphyte	Gymnosperm	Pinaceae	Pinus halepensis	Schiller1986, Conkle1988, Teisseire_1995, Bucci_1998, Agundez_1999, Korol_2002, Gomez_2005	
plant	Spermaphyte	Gymnosperm	Pinaceae	Pinus leucodermis	Boscherini_1994, Bucci_1997	
plant	Spermaphyte	Gymnosperm	Pinaceae	Pinus nigra	Nikolic_1983, Aguinagalde_1996, Cengel_2000, Tolun_2000, AfzalRafii_2007	
plant	Spermaphyte	Gymnosperm	Pinaceae	Pinus pinaster	Vendramin_1998, Salvador_2000, Ribeiro_2001, 2002a, Wahid_2004, Gomez_2005, Bucci_2007	
plant	Spermaphyte	Gymnosperm	Pinaceae	Pinus pinea	Fallour_1997, Vendramin_2008	
plant	Spermaphyte	Gymnosperm	Pinaceae	Pinus sylvestris	PrusGlowacki_1994a, Soranzo_2000, PrusGlowacki_2003, RobledoArnuncio_2005	
plant	Spermaphyte	Monocotyledone	Colchicaceae	Androcymbium gramineum	CaujapeCastells_2003	
plant	Spermaphyte	Monocotyledone	Orchidaceae	Anacamptis palustris	Cozzolino_2003	
plant	Spermaphyte	Monocotyledone	Orchidaceae	Serapias vomeracea	Pellegrino_2007	
plant	Spermaphyte	Monocotyledone	Poaceae	Bromus hordeaceus	Ainouche_1999	
plant	Spermaphyte	Monocotyledone	Poaceae	Lolium perenne	Balfourier_1998	
plant	Spermaphyte	Monocotyledone	Poaceae	Lolium rigidum	Balfourier_1998	
plant	Spermaphyte	Monocotyledone	Poaceae	Melica ciliata	Tyler_2004	
